# The Evolving Landscapes of Invisible Invaders: Global Insights on Plant Viruses and Vector Complexes

**DOI:** 10.3390/v18101144

**Published:** 2026-10-15

**Authors:** Shweta Kumari, Pratap Adinath Divekar, Suhas Gorakh Karkute, Devraj, Arvind Nath Singh, Rajesh Kumar

**Affiliations:** 1 Division of Crop Protection, ICAR-Indian Institute of Vegetable Research, Varanasi 221305, Uttar Pradesh, India; 2 Division of Crop Protection, ICAR-Vivekananda Parvatiya Krishi Anusandhan Sansthan, Almora 263601, Uttarakhand, India; 3 Division of Crop Improvement, ICAR-Central Institute for Cotton Research, Nagpur 440010, Maharashtra, India

**Keywords:** plant viruses, emerging plant diseases, re-emerging viruses, insect vectors, virus detection, virus management

## Abstract

Plant viral diseases remain an important constraint to agricultural production in many parts of the world. In recent decades, emerging and re-emerging viral pathogens have gained prominence, with increasing reports across a wide range of cropping systems. The rapid spread of these viruses is driven by factors such as globalization, expansion of international trade, intensive monocropping, and changing climatic conditions. In many pathosystems, insect vectors such as whiteflies, aphids, thrips, and leafhoppers play a central role in dispersal by facilitating virus transmission between hosts and across geographical boundaries. The epidemiology of viral diseases is influenced by complex interactions among the virus, vector, host plant and environmental factors. Climate-driven shifts primarily in temperature can affect vector dynamics, host susceptibility, and virus accumulation, thereby intensifying the frequency and severity of viral outbreaks. The genetic diversity and evolutionary flexibility of plant viruses further contribute to their capacity to adapt to new hosts and overcome previously effective resistance sources deployed in cultivated varieties. Members of the genera *Polerovirus*, *Crinivirus*, *Fabavirus*, *Trichovirus*, *Tobamovirus*, *Begomovirus*, and *Orthotospovirus* are prominent emerging and re-emerging pathogens reported worldwide and continue to attract attention because of their economic significance. This review provides a global overview of emerging plant viruses, their epidemiology, and advances in detection, transmission, and management strategies aimed at mitigating future virus-induced crop losses.

## 1. Introduction

Being sessile organisms, plants are incessantly exposed to a plethora of microorganisms, including both beneficial and pathogenic invaders. Pathogenic microorganisms cause different kinds of diseases in plants, which drastically affect the growth and development of plants, leading to significant yield losses and making the produce unfit for human consumption. Among various plant pathogens, viruses are particularly formidable “invisible invaders” due to their small genomes, rapid rate of evolution, and extraordinary adaptability. Unlike other diseases, viruses depend upon their host’s cellular machinery for replication, multiplication, movement, and transmission. In response to viral invasion, plants employ multiple defense mechanisms, including RNA silencing, R-gene-mediated resistance, autophagy, and the ubiquitin–proteasome system. Nevertheless, viruses have evolved sophisticated counter-defenses, most notably viral suppressors of RNA silencing, which allow them to evade host immunity. In recent years, the increasing frequency of emerging and re-emerging plant viral pathogens has drawn significant attention due to their expanding geographic distribution, host range shifts, and escalating economic impact. Emerging plant diseases can be defined as those diseases caused by new or reappearing pathogens which, due to their intrinsic characteristics, have the ability to spread rapidly and cause epidemics in certain agro-climatic contexts. Whereas re-emerging viruses are previously known pathogens that were once controlled but have reappeared and regained importance [1]. Such resurgences can arise from factors including the breakdown of host resistance, changes in crop management practices, development of insecticide resistance in vectors, or the widespread cultivation of susceptible varieties.

More than 2100 plant virus species are formally recognized by the International Committee on Taxonomy of Viruses (ICTV) [2]. A large number of plant viruses are transmitted by phloem-feeding hemipteran insects such as aphids, whiteflies, planthoppers, and leafhoppers. Notably, viruses account for more than 47% of all pathogens responsible for emerging and recurrence of plant disease epidemics worldwide [3]. It is estimated that annual crop losses due to plant viral diseases exceed USD 30 billion, posing a serious threat to global food security and agricultural sustainability [4,5]. As agriculture intensifies to meet the demands of a growing human population, the economic burden of plant viral diseases continues to escalate. Furthermore, globalization and the international trade of planting materials have facilitated the long-distance spread of viruses and their vectors into new crops and regions. This has contributed to the emergence and re-emergence of viral pathogens. In parallel, climate change affects plant disease dynamics by altering host susceptibility, vector populations, and crop distributions, resulting in increased selection pressure and a shifting landscape of plant viral diseases [6]. Given the surging risk posed by emerging plant viruses, a deeper understanding of virus–host interactions, transmission mechanisms, and epidemiological factors is essential to developing effective and sustainable disease management strategies. The increasing emergence of viruses is driven by the complex interplay of epidemiological, ecological, molecular, diagnostic, and regulatory factors. Consequently, the present review adopted a systems-level approach to illustrate how these interconnected components collectively influence virus emergence, spread, detection, and management. This review provides a comprehensive overview of emerging and re-emerging plant viruses on a global scale along with their economic importance, molecular mechanisms of transmission, recent advances in detection technologies and novel management strategies.

## 2. Evolving Landscapes of Emerging Viruses: A Global View

In the past decades, distribution patterns of emerging and re-emerging plant viruses have greatly increased. The rationale for selecting the emerging virus were primarily based on published literatures of expanding geographic distribution, increasing incidence and recurrence of outbreaks, in diverse agro-climatic conditions. These viruses belong to diverse taxonomic groups, including members of the genera *Polerovirus*, *Crinivirus*, *Tobamovirus*, *Fabavirus*, *Trichovirus*, *Begomovirus*, and *Orthotospovirus* [7,8,9,10,11]. Their ubiquitous prevalence has been widely reported across all five continents, spanning various cropping systems (Figure 1). For example, tomato brown rugose fruit virus (ToBRFV), which was first identified in the Middle East in 2014, rapidly expanded throughout several continents within a decade and is now a major threat to global tomato production. Outbreaks in Florida are estimated to cause annual economic losses of more than USD 262 million. It can diminish marketable yield losses by 30 to 70 percent in tomatoes [12]. This rapid global expansion highlights the vulnerability of modern agricultural systems to emerging viruses, thereby posing a significant challenge to the achievement of sustainable development goal 2 (SDG 2, Zero Hunger).

### 2.1. Begomovirus Solanumdelhiense (Tomato Leaf Curl New Delhi Virus, ToLCNDV)

ToLCNDV, a bipartite begomovirus has emerged as a major constraint to vegetable-based agroecosystems worldwide over recent decades [13]. ToLCNDV’s host range spans over 90 plant species across 21 botanical families, including a broad array of cultivated and non-cultivated plants (for details, see [14]). As shown in Figure 2, ToLCNDV infects diverse plant groups, such as vegetables, fruits, spices, oilseeds, ornamentals, medicinal and aromatic plants, fiber crops, pulses, and weeds. Among these, vegetables comprise the largest category, about 40% of reported hosts, spanning nine families: Malvaceae, Cucurbitaceae, Solanaceae, Amaranthaceae, Umbelliferae, Fabaceae, Euphorbiaceae, Asteraceae and Apiaceae. Weeds constitute the second-largest group (~23%), followed by ornamental plants (~12%); together, these noncrop hosts represent over one-third of the known host range (Figure 2), and such plants can serve as reservoirs maintaining the virus between cropping cycles and facilitating spillovers at the interfaces of agricultural and natural ecosystems. However, their lower proportion compared with vegetables indicates that ToLCNDV persistence is largely sustained within cultivated crop systems. Other host plants, including oilseed/industrial crops, pulses, fruits, and medicinal plants, each contribute ~6% of the total host spectrum, while fiber crops (~2%) and spices/condiments (~1%) represent minor fractions, suggesting sporadic spillover rather than stable host adaptation (Figure 2).

Originally endemic to the Indian subcontinent, ToLCNDV’s spread in Asia was driven by intensive vegetable cultivation, year-round availability of susceptible hosts, and widespread presence of its vector (*Bemisia tabaci*). The transcontinental dissemination of ToLCNDV beyond Asia is thought to have occurred primarily through the movement of infected planting material, and global trade [13,15]. To date, the virus has been reported from three continents, with the highest number of records from Asia (17), followed by Europe (6) and Africa (4), while no confirmed reports are available from Oceania or the Americas (Figure 3). The predominance of reports from Asia aligns with the virus’s origin and long-term presence in regions with intensive vegetable cultivation that favor continuous vector activity. Conversely, reports from Europe reflect more recent establishment mainly associated with protected cultivation, whereas fewer records from Africa may indicate limited spread or under-detection.

### 2.2. Orthotospovirus Tomatomaculae (Tomato Spotted Wilt Virus, TSWV)

Tomato spotted wilt virus (TSWV), a member of the genus *Orthotospovirus* in the family *Bunyaviridae*, is one of the most widely distributed and destructive plant viruses reported globally [16]. It has an extensively broad host range, with infections documented in more than 1000 plant species across over 95 botanical families. The reported host spectrum is dominated by ornamental plants (425 species) and weeds (415 species), which together represent more than 80% of all known TSWV hosts, with each contributing approximately 42% and 41%, respectively. On the other hand, vegetable crops represent a much smaller portion of the reported host range, accounting for approximately 7% (69 species) (Figure 2). Although vegetables form a relatively small proportion of the total host range, their significance in agriculture and the economy is considerable. TSWV infects several economically important vegetable crops, particularly within the Solanaceae, Cucurbitaceae, and Fabaceae families, where disease outbreaks may lead to considerable yield and quality losses. Other reported host categories include forage and fodder crops (~2%), medicinal and aromatic plants (~4%), spices and condiments (~1%), fruits (~1%), and pulses (~1%). As shown in Figure 2, weeds and ornamental plants appear in almost equal proportions among TSWV hosts, indicating their crucial role in the virus’s survival during the off-season. Ornamental plants may play a significant role in the epidemiology of TSWV, as they are cultivated throughout the year and offer a conducive environment for virus replication and spread [17,18]. At the family level, the Asteraceae contain the highest number of reported TSWV host species, followed by the Solanaceae and the Fabaceae. Weed hosts serve as a crucial ecological reservoir, especially when situated near agricultural fields or within production systems. The high representation of Asteraceae is epidemiologically relevant, as this family encompasses both ornamental plants and weeds. Such overlap can reduce the ecological barrier between cultivated and non-cultivated plants and provide a continuous host bridge that allows for seamless movement across different host environments. This uninterrupted host connectivity creates opportunities for TSWV to persist between cropping cycles and spill over into susceptible crops when suitable vectors and hosts are present. Their presence can maintain virus inoculum, support vector populations, and increase the chance of recurrent infection in cultivated crops. Globally, TSWV has been reported in at least 20 countries in Asia, 19 in the Americas, 34 in Europe, and 4 in Africa (Figure 3). Europe exhibits the widest geographical distribution, followed by Asia, while Oceania has the fewest reported incidences. The presence of TSWV in more than 70 countries across four continents indicates that it is no longer a re-emerging pathogen but a globally established pathogen.

### 2.3. Tobamovirus Fructirugosum (Tomato Brown Rugose Fruit Virus, ToBRFV)

Tomato brown rugose fruit virus (ToBRFV), a member of the genus *Tobamovirus* (family *Virgaviridae*), was first reported in tomato in Jordan in 2014 [19]. Since the initial detection, the virus has been reported in 51 countries distributed across five continents, illustrating the rapid global spread of a newly discovered plant virus (Figure 3 and Supplementary Table S1). Previous studies indicated that ToBRFV infects over 40 plant species belonging to four families, namely, Amaranthaceae, Apocynaceae, Asteraceae, and Solanaceae [20]. Presently, the known host range has expanded considerably and now includes 81 plant species representing more than 20 botanical families (Supplementary Table S1). Weeds account for approximately 69% of known hosts, whereas vegetable crops and ornamental plants comprise 26% and 5% respectively. Despite this apparent diversity, tomato and pepper remain the only crop species that have been confirmed as natural hosts under field conditions [21]. An examination of weed-associated reports further shows that only about 25% correspond to naturally infected species distributed across 12 families, whereas the remaining 75% represent experimentally susceptible hosts belonging to 17 families that were identified under controlled conditions (for species details, see [20]). These observations indicate that ToBRFV possesses a broad adaptive capacity for host association under experimental conditions, although the ecological significance of many of these hosts under natural conditions remains to be fully understood. Geographically, ToBRFV has been reported in 14 countries in Asia, 28 in Europe, 6 in the Americas, 2 in Africa, and 1 in Australia. Europe shows the greatest diversity of ToBRFV occurrences, followed by Asia, whereas Oceania has the lowest (Figure 3). The relatively high number of reports from Europe may reflect a combination of intensive surveillance and strict phytosanitary monitoring. In contrast, lower numbers reported from some regions may be influenced by limited surveillance intensity, diagnostic infrastructure and crop distribution patterns. Overall, these patterns establish ToBRFV as a globally emerging plant virus of serious concern, underscoring the need for enhanced surveillance and strict phytosanitary measures to mitigate its potential impact on agricultural productivity and food security.

### 2.4. Trichovirus Pinovitis (Grapevine Pinot Gris Virus, GPGV)

Grapevine pinot gris virus (GPGV), an emerging member of the genus *Trichovirus* within the family *Betaflexiviridae*, has been increasingly reported from grape-growing regions worldwide. At present, the virus is associated with 24 reported plant hosts worldwide, with *Vitis vinifera* serving as the primary host. Additionally, the virus has been detected in 12 weed species including *Ailanthus*, *Asclepias*, *Crataegus*, *Fraxinus*, *Rosa*, *Rubus*, *Sambucus*, *Chenopodium album*, *Silene latifolia*, *Vitis californica*, *V. coignetiae*, and various *Vitis* hybrids (Supplementary Table S1). Weeds account for approximately 50% of reported hosts, whereas medicinal and aromatic plants contribute about 25% and ornamental plants nearly 21% (Figure 2). To date, there have been no reports of GPGV infecting vegetable crops in any region. The virus was first identified in Italian grapevines exhibiting stunted growth and chlorotic mottling symptoms and classified as grapevine leaf mottling and deformation (GLMD) disease [22]. Following its initial discovery, characterization using small RNA high-throughput sequencing enabled a more detailed understanding of the virus and led to its name GPGV [23]. Since then, the virus has been documented in more than 42 countries distributed across all five continents [24]. Available reports indicate that Europe has the highest number of documented occurrences, with records from 25 countries, followed by the Americas with 8 countries and Asia with 7 countries (Figure 3). In contrast, GPGV detection in Africa and Oceania is limited to a single country on each continent, suggesting that these regions have either low incidence or inadequate surveillance. Furthermore, differences in surveillance intensity, vineyard monitoring programs, diagnostic availability, and reporting practices may influence the number of published records. Nevertheless, currently available data indicate that Europe remains the region where GPGV has been the most extensively reported. Phylogeographic studies have suggested that GPGV may have originated in Asia, possibly in China, and has spread globally through the international movement of infected grapevine propagative material [25]. In terms of impact, GPGV infections have a substantial impact on grape production. Yield reductions of up to 50% have been reported, largely due to decrease in both grape cluster number and weight [26,27]. The widespread global distribution of GPGV across many grape-producing regions underscores the necessity of continuous vineyard surveillance and robust grapevine certification programs to provide virus-free planting materials. Reliable detection methods and the production of virus-free planting materials remain essential components of disease prevention because infected vines may act as long-term reservoirs that facilitate dissemination of the virus.

### 2.5. Fabavirus Betaviciae (Broad Bean Wilt Virus 2, BBWV-2)

Broad bean wilt virus (BBWV), a member of the genus *Fabavirus* (family Secoviridae), is recognized as a widely distributed and globally damaging pathogen, infecting more than 200 plant species across 41 botanical families [28]. The virus comprises two most important species, Broad bean wilt virus 1 (BBWV-1) and Broad bean wilt virus 2 (BBWV-2) [29]. Of these, BBWV-2 has received considerable attention because of its broad host range and recurrent association with disease in economically important crops. BBWV-2 has been reported to infect more than 50 plant species belonging to 27 families. Based on documented host records, ornamental plants represent the largest host category, accounting for approximately 39% of reported hosts. Vegetable crops form the next major category, contributing nearly 33%, including 16 species. Medicinal and aromatic plants represent around 12%, weeds 14%, and oilseed crops approximately 2% of reported hosts (Figure 2). The global distributions of BBWV-1 and BBWV-2 show distinct patterns. BBWV-1 has so far been reported in Europe and North America, whereas BBWV-2 has a wider distribution and has been documented in both temperate and subtropical regions worldwide [28]. Available records show BBWV-2 occurrence in nine Asian countries, seven European countries, two countries each in America and Oceania, and one country in Africa (Supplementary Table S1; Figure 3). Across continents, Asia, particularly China, represents a major hotspot of BBWV-2 diversity. Repeated occurrence in China, followed by Korea, suggests that the virus is well established in parts of East Asia and may pose a major threat to economically important cultivated crops in those regions.

### 2.6. Cucurbit Aphid-Borne Yellows Virus (CABYV), Genus Polerovirus

Poleroviruses were formerly classified within the luteovirids and were reassigned to the family *Solemoviridae* in 2021 [2]. The genus *Polerovirus* has expanded rapidly in recent years, with more than 100 plant species recorded as natural hosts. Notably, crops belonging to the Cucurbitaceae and Poaceae families appear to be particularly affected by polerovirus infections. Cucurbit aphid-borne yellows virus (CABYV), an emerging member of the genus *Polerovirus*, has been reported to infect at least 31 plant species worldwide. Among these hosts, vegetables account for approximately 68%, whereas weeds represent about 23%. Pulses, oilseed crops, and fruit crops each contribute approximately 3%, with one species reported in each category (Figure 2, Supplementary Table S1).

CABYV was first identified in France in 1988 in melon, cucumber, and zucchini [30]. Since its first report, the virus has not remained restricted to Western Europe; within two decades, its presence had been documented across multiple continents [31,32]. By 2020, 26 *polerovirus* species had been formally recognized by the International Committee on Taxonomy of Viruses [2]. Over the subsequent three years, this number increased substantially, with 51 additional species being incorporated into the official taxonomy [33]. In addition, nine new tentative *Polerovirus* species have been reported more recently, including *Parsley polerovirus*, *Cynanchum yellow mottle-associated virus*, *Rice dwarf virus, Bitter gourd yellowing crumple virus, Carrot polerovirus 1, Cacao polerovirus, Cacao leaf roll virus, Bitter apple aphid-borne yellow virus*, and *Viola philippica polerovirus* (Supplementary Table S1). This rapid increase in species is driven largely by frequent recombination events both within the genus and between viruses belonging to different families [34,35]. Poleroviruses are now reported in all continents, although their documented diversity differs among regions. Asia shows the highest reported diversity, with 51 species from 17 countries, followed by the Americas, with 22 species from 4 countries; Africa, with 17 species from 16 countries; Europe, with 16 species from 8 countries; and Oceania, with 14 species from 2 countries (Figure 3). Among the 77 total Polerovirus species reported worldwide, Oceania shows the lowest diversity, limited mostly to Australia and Papua New Guinea (Figure 3; Supplementary Table S1). The occurrence of CABYV in 47 countries over four decades highlights its remarkable adaptive potential and the dynamic nature of its spread. Frequent recombination, diversification, and efficient vector transmission further reinforce its status as a highly threatening pathogen to cucurbit cultivation globally.

### 2.7. Crinivirus

Criniviruses (family Closteroviridae) have a broad host range, infecting more than 230 plant species representing at least 40 families, including numerous cultivated crops and weed species. Currently, 14 *Crinivirus* species are officially recognized by the ICTV (https://ictv.global/taxonomy, accessed on 20 March 2026). Several additional tentative species, including *Strawberry Kurdistan virus, Strawberry crinivirus 4*, and *Mulberry crinivirus*, have recently been reported in countries such as Iran, Canada, and China (Supplementary Table S1). Among the recognized species, *Cucurbit yellow stunting disorder virus* (CYSDV), *Cucurbit chlorotic yellows virus* (CCYV), *Tomato chlorosis virus* (ToCV), and *Lettuce chlorosis virus* (LCV) account for nearly 90% of documented crinivirus infections worldwide. Comparative analysis of these four viruses indicates notable differences in both host range and geographic distribution. Among them, ToCV has the largest host range, with approximately 131 reported host species and records from about 46 countries. In contrast, CYSDV, CCYV, and LCV have been reported in fewer host species (Figure 4; Supplementary Table S1). Initially CYSDV and CCYV were largely restricted to cucurbit hosts [36]. Subsequent studies, however, have expanded the known host range of these viruses, with CYSDV reported in at least 11 plant families and CCYV in at least 14 families. Despite this broader host spectrum, cucurbits continue to represent the primary hosts of both viruses, with limited spillover into non-cucurbit species, particularly legumes. It is noteworthy that ToCV has dramatically expanded its host range from infecting 24 species in 7 families reported in 2006 [37] to 131 plant species in 33 families at present (Supplementary Table S1). Across these major criniviruses, weed hosts consistently outnumber cultivated hosts, highlighting their role as a key driver of crinivirus evolution rather than incidental hosts. Notably, the broader host range and wider distribution of ToCV likely increase opportunities for diversification and the emergence of novel strains, whereas the narrower host ranges of CYSDV, CCYV, and LCV may impose evolutionary constraints on these viruses.

## 3. Symptomatology and Economic Importance

Plant viruses induce a wide spectrum of symptoms, such as chlorosis, mosaic patterns, leaf deformation, necrosis, stunting, and fruit abnormalities. Symptom severity depends on several factors, including host genotype, age of the plant, viral strain and environmental conditions. Symptom-based diagnosis is essential to the accurate identification of diseases and the timely implementation of interventions, which are crucial to preventing disease progression and spread to new hosts or regions. Typically, *Cucurbit aphid-borne yellows virus* (CABYV) infection in cucurbits is characterized by yellowing, interveinal chlorosis, leaf thickening and brittleness [38]. Association of viruses in cultivated crops not only affects produce quality but also diminishes total marketable yield. Such economic losses have been reported worldwide in several crops, ranging from negligible to complete failure. For instance, yield losses due to CABYV reach up to 40–50% in zucchini, pumpkin and cucumber; 40–70% in groundnut; and 70% in bitter gourd [39,40,41,42]. Furthermore, the number of fruits per plant and overall yield are diminished by 51% and 40% respectively, due to high rates of flower abortion in cucumber infected by CABYV [30]. Symptoms of criniviruses such as CYSDV and CCYV are characterized by diffuse chlorotic mottling, interveinal chlorosis, leaf thickening, and brittleness especially on older leaves [9]. These yellowing symptoms are often mistaken for nutrient deficiencies, senescence, or phytotoxicity, which can hinder timely and accurate field diagnosis. Crinivirus infections cause significant yield losses ranging from less than 1% to 63%, depending on the host and infection stage [9,43]. For example, CCYV causes severe yield losses from 60 to 80% in melon, squash, and watermelon and of ~20% in cucumber [43]. Similarly, CYSDV has been associated with 30–50% yield losses in cucumber and up to 80% in melon [44,45,46]. Leaf curling, yellow mosaic and stunting symptoms are commonly associated with the tomato leaf curl new delhi virus (ToLCNDV) [47]. In some cases, infection can cause roughness and longitudinal cracking on the fruit surface, which greatly impairs fruit quality. It causes yield losses of over 20% in zucchini and melon and 80% in pumpkin in the Mediterranean region [48,49,50]. In the Indian subcontinent, it reaches up to 100% in sponge gourd and complete crop failure in tomato when infection occurs at an early stage [51,52]. Recently, ToLCNDV outbreaks in China affected an area of ~650 ha of cucurbit cultivation and caused losses of ~USD 15 million [53].

*Tomato spotted wilt virus* (TSWV) typically causes chlorotic or necrotic lesions on leaves, stem and fruits, along with vein necrosis and flower malformation. TSWV infection causes yield losses of 42 to 95% in tomato and 40 to 60% in peanut [54]. *Broad bean wilt virus 2* (BBWV-2) infection leads to mosaic, vein yellowing, wilting, leaf distortion and stunting [28]. *Tomato brown rugose fruit virus* (ToBRFV) causes distinctive symptoms including mosaic, mottling, brown rugose patches, green streaks, and the uneven ripening of fruits [55]. Fruit weight and yield losses due to ToBRFV infection are diminished by 25–40% and 15–55% respectively, in tomato crops harboring the *Tm-22* resistance gene [56]. Notably, many weed hosts can remain asymptomatic despite the presence of virus load, differing in their efficiency in transmitting viruses to crop plants [57]. Altogether, viral infection has a significant impact on crop productivity by impairing photosynthesis, reducing fruit set and ultimately diminishing economic yields. Additionally, their overlapping symptomatology complicates accurate diagnoses and timely management interventions, thereby amplifying economic losses.

## 4. Molecular Determinants of Virus Transmission

Effective management of viral plant diseases requires an understanding of how viruses move within and between host plants. Plant viruses disseminate through various mechanisms, including insect vectors, mechanical, grafting, and seeds or vegetative propagation (Figure 5). Among them, insect-mediated transmission is by far the most common, accounting for approximately 80% of known plant viruses [58]. Among these insect-transmitted viruses, nearly 55% are transmitted by hemipteran insects such as whiteflies, aphids, mealybugs, planthoppers, and leafhoppers [59,60]. Whitefly-transmitted viruses represent one of the largest groups threatening global agriculture. Whitefly (*Bemisia tabaci*), a cryptic species complex often called a “supervector,” can transmit more than 300 different plant viruses [61], making it one of the most important quarantine pests worldwide. Insect transmission of plant viruses generally follows either of the two paradigms. In the first, a viral helper component (HC) protein acts as a molecular bridge, linking the viral coat protein to a receptor in the vector; whereas in the second, a viral protein directly binds a receptor in the vector [62].

### 4.1. TSWV (Tomato Spotted Wilt Virus)

TSWV is transmitted in a persistent, propagative manner by the western flower thrips (*Frankliniella occidentalis*). The TSWV genome encodes five proteins (RdRP, NSm, Gn and Gc, NSs, and N), of which the nucleocapsid protein (N) and glycoproteins (Gn and Gc) are essential to virion forming and vector interaction [18]. During feeding, thrips secrete elicitor protein (FoCSP1), which is perceived by plant receptors. Upon recognition, plants activate jasmonic acid (JA)-mediated defense pathways that reduce vector fitness and fecundity [63,64,65]. To counteract, TSWV induces the salicylic acid (SA) signaling pathway, which suppresses JA-responsive genes via its NSs protein [66]. The signaling crosstalk between the SA-JA-mediated pathways diminishes the impact of plant defenses on thrips without altering the vector’s feeding behavior [66]. After acquisition, virus entry into the midgut is mediated by interaction between viral glycoproteins and receptor protein (Fo-GN) of midgut cells through a receptor-mediated endocytosis mechanism [67,68]. After entry, the virus exploits host factors like Fo-Cyp1 protein, which can suppress thrips’ antiviral responses to facilitate viral replication, while other proteins, such as obstructor-E-like protein (Obst), enhance virus acquisition and transmission efficiency [69]. After replication, TSWV moves farther from the midgut to the salivary glands, where it accumulates and becomes transmissible. The underlying mechanisms by which TSWV move further from the midgut to the salivary glands before transmission remain poorly understood.

### 4.2. Poleroviruses and Their Vectors

Poleroviruses are phloem-limited viruses primarily transmitted by aphids in a persistent, circulative, non-propagative manner [70]. Recent studies described that whiteflies can also transmit a few poleroviruses, such as pepper whitefly-borne vein yellows virus and cucurbit whitefly-borne yellows virus, indicating a potential shift or expansion in vector specificity within the genus [71,72]. The interactions among host, vector and polerovirus are complex and can affect vector fitness positively, negatively, or neutrally [73,74,75,76]. In the genus *Polerovirus*, three viral proteins, viz., P0, P1, and P7, play a crucial role in subverting plant defense pathways. However, the detailed mechanisms of signaling crosstalk and their impact on vector fitness remain unclear. Additionally, the molecular identity and mechanism of host receptors involved in host–virus–vector interactions are still unknown, representing a significant gap in our understanding of virus transmission and an obstacle to devising strategies to curtail virus spread.

### 4.3. Criniviruses and Whitefly Transmission

Criniviruses (family *Closteroviridae*) have bipartite, positive-sense and single-stranded RNA genomes [77,78]. The RNA1 genome encodes RNA-dependent RNA polymerase (RdRP), while RNA2 encodes five key proteins, namely, major coat protein (CP), minor coat protein (CPm), a heat shock protein 70 homolog (HSP70h), P6, and P59 [79]. Among these, CPm was identified as the primary molecular determinant in vector transmission, facilitating virion attachment to the vector’s receptors present in the cibarium region of the foregut [80,81]. Thus, unlike TSWV (which uses a helper component protein), criniviruses rely on a capsid-based mechanism involving direct virion attachment to the vector’s cuticle. However, the receptor proteins involved in the perception and mechanisms underlying this attachment remain to be explored. Criniviruses such as CCYV and CYSDV are transmitted by cryptic *B. tabaci* species (e.g., MEAM1 and MED) in a semi-persistent manner [36,82,83].

### 4.4. Begomoviruses and Their Vectors

Begomoviruses (family *Geminiviridae*) are transmitted by *Bemisia tabaci* (whiteflies) in a persistent and circulative manner. However, whether the transmission is non-propagative or propagative remains under discussion [84,85]. After acquisition, viruses need to overcome the insect’s midgut barrier, circulate through the hemolymph, and ultimately reach the salivary glands before being inoculated into a new plant [86]. At the molecular level, begomoviruses manipulate insect signaling pathways (JAK/STAT) to maintain equilibrium between viral persistence and vector fitness [87]. Simultaneously, begomovirus infection alters host plant physiology by suppressing the JA signaling pathway, which reduces anti-insect compounds (terpenoids). This whole process is facilitated by specific interactions between viral coat protein (AV1) and insect proteins, which are crucial to stabilizing virions and enabling their transport through the insect’s body [88,89]. As begomovirus and crinivirus often share hosts and vectors, co-infections are common under natural conditions. Such mixed infections can lead to synergistic effects, altering disease severity and increasing epidemic risk [90,91].

### 4.5. Other Viruses and Transmission Modes

ToBRFV is predominantly transmitted mechanically; however, long-distance dissemination occurs via contaminated seeds and fruits [21]. No insect vector has been conclusively identified for ToBRFV thus far. Nonetheless, bumblebees (*Bombus terrestris*) have been shown to act as mechanical carriers of virus-infected pollen under greenhouse conditions [92]. Conversely, *Grapevine pinot gris virus* (GPGV) spreads primarily through the movement of infected propagation materials on regional and global scales [93,94]. Within vineyards, its local spread is facilitated by the eriophyid mite *Colomerus vitis*, which is presently the only confirmed vector of GPGV [95,96]. In addition, transmission may occur via agricultural practices such as pruning and grafting [97]. *Broad bean wilt virus 2* (BBWV-2), in contrast, is transmitted by aphids (*Aphis gossypii* and *Myzus persicae*) in a non-persistent manner, with high transmission efficiency varying from 60 to 90% under controlled conditions [28]. The BBWV-2 movement protein (VP37) acts as an RNA-silencing suppressor [98]. However, the molecular basis of virus’s etiology, pro-viral factor and its interactions with hosts remain largely unknown.

## 5. Seed Transmission

Seed transmission represents a critical route for the persistence and long-distance spread of many plant viruses. Approximately 18–20% of known plant viruses can be transmitted through seeds [99,100]. Among emerging viruses, *Tomato brown rugose fruit virus* (ToBRFV) is a seed-borne pathogen and contaminated seeds are a major source of its global dissemination [101]. Notably, seeds from infected tomato fruits showed up to 100% contamination, though the virus is mostly confined to the outer seed coat (testa) rather than the embryo [102]. Despite having low seed-to-seedling transmission rates (~0.1–2.8%), infected seeds can act as a source of primary inoculum that leads to secondary spread within fields or new regions [102]. Recent research reported seed-to-seedling transmission of ToBRFV in the weed *Solanum nigrum*, with a transmission rate of approximately ~1.9%, suggesting that weed reservoirs could also contribute to virus persistence [103]. Furthermore, ToBRFV has been detected in reproductive tissues and pollens, but not in ovules, indicating that its transmission via pollen is unlikely [104]. Seed transmission has also been reported for TSWV, although results vary across different hosts and studies [105,106]. Furthermore, seed transmission has been reported recently for several begomoviruses, including *Sweet potato leaf curl virus, Mungbean yellow mosaic virus, Tomato yellow leaf curl virus, Bitter gourd yellow mosaic virus, Dolichos yellow mosaic virus*, and ToLCNDV [107]. Noteworthy, viruses in the genus Fabavirus (such as BBWV) are not known to be seed-transmitted yet [108]. These observations underscore how seeds can serve as a significant pathway for spreading viruses across time and space.

## 6. Determinants of Virus Emergence: A Comprehensive View

The emergence of plant viral diseases is determined by the complex interplay of molecular and ecological factors that extend beyond the traditional disease triangle concept. In vector-borne systems, this concept is better described as a disease tetrahedron with the vector as a fourth essential component [109]. Within this conceptual framework, temperature is a key environmental determinant that influence host–virus interactions, systemic movement and transmission efficiency (Figure 6a). Elevated temperatures have been shown to increase viral accumulation, systemic movement, and disease severity in several plant–virus systems [110,111]. However, the relationship between temperature and viral accumulation is not linear and varies among host–virus interactions. For example, in some pathosystems, higher temperatures reduce symptom severity by enhancing RNA-silencing pathways [112,113,114,115,116,117], whereas in other cases, it promotes disease progression by disrupting RNA-silencing pathways [118,119]. These contrasting observations indicate that temperature-mediated effects are highly context-dependent and influenced by host genotype, viral strain, and host cellular factors essential to viral replication. The host genetic factors at the molecular level, particularly resistance (R) genes and susceptibility (S) factors, play a decisive role in viral emergence. R-gene-mediated resistance gets suppressed with the elevation in temperature and thereby enhances susceptibility, subsequently triggering disease emergence [115,116]. Notably, several plant viruses, such as TSWV and ToBRFV, have evolved resistance-breaking (RB) strains which overcome host R-gene-mediated resistance [120,121]. Thus, a balance between resistance mechanisms and susceptibility determinants critically shapes disease outcomes and viral emergence (Figure 6b).

Vector biology constitutes another key determinant in the emergence of plant viruses. Insect vectors such as aphids, whiteflies, and thrips are highly sensitive to environmental cues, particularly temperature and humidity. Climate warming accelerates vector development, enabling multiple life cycles within a single season and enhancing transmission efficiency [122,123]. Elevated temperatures can enhance virus transmission by facilitating the expansion of insect vectors into new geographical areas (Figure 6b). For example, rising temperatures have caused the spread of *Papaya ringspot virus* (PRSV) into regions of India [124] and the expansion of *Potato virus Y* (PVY), *Tomato yellow leaf curl virus* (TYLCV) and *Barley yellow dwarf virus* (BYDV) in Europe [6]. Further increased temperatures have also been associated with increased transmission efficiency of viruses like *Soybean mosaic virus* and *Tomato yellow leaf curl virus*, largely due to altered vector feeding behavior [125]. These responses are often nonlinear and species-specific, leading to shifts in vector distribution and invasion into new zones or hosts. Moreover, global trade and human-mediated movement of planting materials facilitate rapid virus dissemination, as evidenced by outbreaks of grapevine Pinot gris virus and other vector-transmitted viruses [95,96,97]. Climate change imposes novel selection pressures that accelerate mutation, recombination, and host range expansion. While long-term host–virus co-evolution in natural ecosystems often results in tolerance [126], environmental perturbations can disrupt this equilibrium, leading to disease outbreak. Despite these advances, key gaps remain. The mechanisms by which temperature modulates host susceptibility and virus–host interactions at the molecular level remain unclear. Furthermore, the limited understanding of viral persistence in non-cultivated reservoirs, their role in host shifts, and climate-driven spillover events remain poorly understood. Addressing these interconnected processes is essential to improving our understanding of viral emergence and for developing sustainable, climate-resilient disease management strategies.

## 7. Advances in Detection of Plant Viruses

Recent advances in diagnostic techniques have substantially improved virus detection speed, sensitivity, and reliability. Due to their cost-effectiveness and ease of use, conventional serological assays such as enzyme-linked immunosorbent assay (ELISA), dot-blot immunoassay (DBIA), and tissue-blot immunoassay (TBIA) remain widely employed for the screening of large biological samples [127]. However, these methods offer limited specificity, restricting their application in field diagnostics. Molecular methods including reverse-transcription PCR (RT-PCR), multiplex RT-PCR, and quantitative RT-PCR (RT-qPCR) offer higher sensitivity and specificity for virus detection [128]. However, these techniques are labor-intensive and require laboratory facilities. In recent years, several techniques such as loop-mediated isothermal amplification (LAMP), recombinase polymerase amplification (RPA), CRISPR-Cas and next-generation sequencing (NGS) methods have revolutionized plant virus diagnostics. These diagnostic platforms have been employed for detection of several economically important and novel viruses [129,130,131,132]. For instance, ToLCNDV can be detected via conventional PCR-based assays, rolling-circle amplification, LAMP, RPA and CRISPR-Cas-mediated techniques [133,134,135,136]. Similarly, criniviruses such as CYSDV and CCYV are routinely detected using traditional immunoassays and RT-PCR or qPCR assays [137,138,139]. CABYV can be detected using ELISA and RT-PCR, whereas high-throughput sequencing (HTS) has recently been employed for its full genomic characterization [129,140]. For viruses causing latent infections (e.g., GPGV), real-time RT-PCR is commonly used for detection, as infections might not show symptoms in some host plants [141]. Likewise, BBWV2 (genus fabaviruses) has been detected by both serological and nucleic acid-based assays [142,143]. Although serological and molecular techniques such as ELISA and RT-PCR remain standard methods for detecting ToBRFV, various advanced techniques, including multiplex PCR, digital droplet PCR and RT-LAMP assays, have been employed for its quick detection [144,145,146].

High-throughput sequencing technologies, particularly the nanopore sequencing approach, facilitate the rapid identification of novel plant viruses, detection of resistance-breaking variants, and comprehensive analysis of viral population diversity within infected plants [147]. For instance, during disease outbreaks, high-throughput sequencing approaches have facilitated the rapid characterization of resistance-breaking strains of TSWV, enabling the identification of mutations associated with the breakdown of host resistance [148,149]. Alongside these advances, recent developments in the CRISPR–Cas system have opened up new avenues for rapid and specific plant virus detection in addition to its well-established applications in genome editing. CRISPR-Cas-mediated platforms utilize the collateral cleavage activity of Cas proteins such as Cas12a, Cas13, and Cas14a, which exhibit collateral cleavage activity upon recognition of the target [150,151]. The most prominent examples include Specific High-sensitivity Enzymatic Reporter unLOCKing (SHERLOCK) and DNA Endonuclease Targeted CRISPR Trans Reporter (DETECTR), which have demonstrated rapid, field-deployable diagnostics of plant viruses with high sensitivity [152]. In a SHERLOCK assay, viral RNA is first amplified by RT-RPA methods; subsequently, the amplified RNA is recognized by a target-specific crRNA–Cas13 complex. On the other hand, in the DETECTR platform, viral DNA or reverse-transcribed viral cDNA is first amplified using isothermal amplification methods like RPA, and the amplified target is recognized by a sequence-specific crRNA-Cas12 complex [152,153,154]. Among the earliest reports of CRISPR-based virus detection in plants was a CRISPR/Cas12a-based one-step, in-field diagnostic assay capable of detecting plant RNA viruses such as *Potato virus X* (PVX), *Potato virus Y* (PVY) and *Tobacco mosaic virus* (TMV) within 30 min [155,156,157]. Alon et al. [158] employed a CRISPR-Cas12a assay to specifically detect ToBRFV and TMV in infected tomato plants. To evaluate the diagnostic performance of different Cas variants for ToBRFV detection in tomato, Besati et al. [159] employed both CRISPR-Cas12a and CRISPR-Cas9 systems in combination with RPA. Their results indicated that both Cas12a- and Cas9-mediated systems enable rapid detection of ToBRFV when coupled with RPA and lateral flow assay (LFA) devices. More recently, artificial intelligence (AI) has been integrated with CRISPR diagnostics to improve gRNA design and assay specificity. An AI-driven pipeline for ToBRFV detection identified conserved viral regions and designed optimized crRNAs for a Cas13a-based assay [160]. However, most CRISPR-based diagnostics platforms rely on pre-amplification of target nucleic acid, which may lead to false positives. To address this, CRISPR-Cas based detection systems without pre-amplification steps are being developed. For instance, Langer et al. [161] developed a highly sensitive amplification-free LbCas12a-Ultra assay with a 7nt stem-loop reporter which showed 10-fold increase in sensitivity compared with LbCas12a with the linear reporter detection assay. These advances mark significant progress toward more reliable, field-ready CRISPR diagnostics. In the context of climate change and rapidly evolving viruses, fast and accurate detection will be crucial to timely plant disease management.

## 8. Management of Viral Diseases

Plant viruses have emerged as major constraint for crop productivity, causing severe losses and threatening global food security. Traditionally, managing plant viral diseases has relied on conventional approaches such as use of resistant varieties, cultural control, and chemical and biological measures to reduce vector populations and virus spread. Among these, development and deployment of genetically resistant cultivars remain the most reliable, sustainable and cost-effective approaches [162]. However, the rapid evolution of viral pathogens and their insect vectors often result in the emergence of strains capable of overcoming such resistance, necessitating continual development of new resistant varieties [163]. Chemical insecticides can effectively reduce vector populations and thus virus transmission. Nevertheless, intensive use of pesticides raises environmental concerns, risks to off-target organisms, and increases the risk of vectors developing insecticide resistance. These limitations highlight the need for more sustainable and durable virus management strategies. Recent advances in biotechnological approaches such as transgenic and non-transgenic approaches have provided promising alternatives to enhance host resistance or disrupt virus–host interactions. Additionally, effective plant health management also depends on rigorous testing, surveillance, and stringent phytosanitary regulations to prevent the introduction and spread of viruses via infected plant materials [162]. Such measures are particularly critical for viruses designated as quarantine pests, where early detection and trade restrictions are used to limit their spread. For example, TSWV, ToLCNDV, and ToBRFV are designated quarantine pests in some regions (listed as A2 quarantine pests by EPPO) [14,20]. Other viruses, such as CABYV, CYSDV, and BBWV-2, are under regional monitoring to minimize their spread through international trade of plant materials. Therefore, integrating point-of-care (POC) diagnostics with robust phytosanitary measures is essential to curbing the global movement of plant viral pathogens.

### 8.1. Transgenic Approaches

Transgenic plants engineered for virus resistance involve the introduction of viral genes in plants, resulting in interference with viral infection, replication or movement. This strategy is referred to as pathogen-derived resistance (PDR). One of the earliest examples of PDR was demonstrated against tobacco mosaic virus in transgenic tobacco plants expressing the viral coat protein [164]. Subsequently, this strategy was extended to include the expression of different viral genes or fragments in various crops to confer strong resistance against a range of plant viruses. Advances in transgenic technologies led to the adoption of RNA-silencing-based approaches, which have been effectively utilized to confer resistance against plant viruses. RNA silencing is a natural plant defense mechanism that detects viral RNAs and degrades them [165]. Like PDR, RNA interference (RNAi) approaches have demonstrated broad success in conferring resistance across numerous crop species against several major plant viruses [165,166,167]. Alternatively, the host-induced gene silencing (HIGS) strategy involves the silencing of genes in viruses by expressing homologous double-stranded small interfering RNAs (siRNAs) in host plants [168,169]. Although pathogen-derived resistance and RNA-silencing-based approaches have effectively conferred virus resistance in plants, various technical, regulatory, and biosafety-related limitations have constrained their widespread adoption.

### 8.2. Non-Transgenic Approaches

Significant attention has recently shifted toward non-transgenic approaches to managing plant viruses, without permanently inserting foreign DNA into plant genomes. Among these, RNA-based methods are applied externally to induce a similar antiviral response. As an example, Spray-induced gene silencing (SIGS) is a novel and eco-friendly approach that can be easily adapted to manage viral diseases [170]. It is also known as environmental RNAi, which refers to direct application of dsRNAs or siRNA onto host plants, which leads to the silencing of the virus gene and results in efficient disease control [171]. Previous studies have demonstrated that topical application of dsRNAs in crops like zucchini, cucumber and papaya can considerably reduce symptom severity and viral loads in infected plants [172]. Studies have also demonstrated that dsRNA-based applications targeting viral genes were effective against at least 14 different positive-sense RNA viruses [173]. It was reported that the SIGS approach can delay symptom development and virus accumulation in host plants infected by negative sense viruses such as TSWV [174,175] and *Groundnut bud necrosis virus* (GBNV) [176]. Advances in nanotechnology-based dsRNA delivery systems have enhanced the stability and uptake of sprayed dsRNAs in plants under field conditions.However, the silencing efficiency of these nanocomplexes remains largely restricted to the local regions of application [177,178,179,180,181]. Sun et al. [181] developed an environmentally friendly, low-cost cationized bovine serum albumin (cBSA) dsRNA nanocomplex platform for efficient systemic gene silencing in tobacco and poplar. Another non-transgenic strategy is cross-protection, where plants are inoculated with mild (attenuated) strains of a virus to protect them from virulent strains of the same virus [182]. Cross-protection has been successfully employed in some crops where resistance genes are not known. However, care must be taken because mild strains may often interact with other viruses or mutate, leading to unexpected disease outbreak or more virulent strains.

Development of host plant resistance is the most important approach for management of diseases. Host plant resistance is characterized primarily by resistance genes. Therefore, mapping such resistance genes or QTLs and developing molecular markers linked to them are key strategies for crop improvement and genetic breeding [183]. Using molecular markers, 226 pepper germplasm accessions were evaluated against nine viral diseases, i.e., TMV, CMV, PVY, TSWV, *Pepper mild mottle virus* (PMMoV), *Chilli veinal mottle virus* (ChiVMV), *Chilli ringspot virus* (ChiRSV), *Tomato mottle mosaic virus* (ToMMV) and BBWV2. Of these, 65 accessions exhibited resistance to one or multiple viruses [184]. Similarly, Zhao et al. [185] established an effective disease evaluation system in tomato for *Tomato chlorosis virus* (ToCV) resistance. Twenty-five tomato varieties were screened using an improved stem-inoculated method, and resistance that can be utilized in tomato breeding programs was found in three varieties. The mapping of QTLs responsible for genetic resistance is also being done by genome-wide association studies. Recently, twelve QTLs associated with CABYV and seven QTLs associated with CMV were mapped in cucumber by a GWAS in 256 elite lines and landraces [186]. Interestingly, they also mapped five QTLs linked to reducing attractiveness for the *Aphis gossypii* vector. These QTLs could be utilized effectively to develop elite cultivars to manage viral diseases in cucumber.

More recently, CRISPR-Cas genome editing has emerged as a powerful tool to develop durable and broad-spectrum resistance against plant viruses by targeting viral genomes or host susceptibility factors [187]. It has been demonstrated that over-expression of Cas9 protein with guide RNAs targeting the viral genomes conferred resistance on *Nicotiana benthamiana*, Arabidopsis and barley against *Wheat dwarf virus*; tomato against *Tomato yellow leaf curl virus*; *Banana against banana streak virus*; and several other crops [188,189,190]. Similarly, Cas variants such as FnCas9 and Cas13a were used to target plant RNA virus genomes to induce virus resistance in plants [191]. This approach has been utilized in *N. benthamiana* to target ssRNA virus TuMV, in potato to target *Potato virus Y* (PVY) and in rice to target *Southern rice black-streaked dwarf virus* (SRBSDV) and *Rice Stripe Mosaic Virus* (RSMV) [192,193,194,195]. Another promising approach is to suppress the negative regulators of disease resistance or host susceptibility genes, which results in broad and durable resistance in plants [196]. A recent study identified *SlDnaJ* (Solyc10g081220) as a key locus regulating TSWV resistance in tomato and characterized the co-chaperone gene *Sldnaj* (structural variant of *SlDnaJ*) carrying a 61 bp promoter deletion specifically associated with TSWV infection in tomato. Mutation in *Sldnaj* TSWV-susceptibility genes can confer enhanced resistance against TSWV, indicating its potential as a promising target for developing TSWV-resistant tomato varieties using CRISPR tools [197]. Furthermore, plant RNA viruses require host factors such as eukaryotic translation initiation factors *(eIFs)* for their replication, and the CRISPR-mediated editing of *eIF* genes in different plant species has been shown to confer strong resistance against RNA viruses. Among the earliest reports, Chandrasekaran et al. [198] targeted *eIF4E* in cucumber and achieved broad-spectrum resistance against *Cucumber vein yellowing virus* (CVYV), *Papaya ringspot mosaic virus*-w (PRSV-W), and *Zucchini yellow mosaic virus* (ZYMV). The other reports include resistance against *Turnip mosaic virus* (TuMV) in Chinese cabbage by editing the *eIF*(*iso*)*4E* gene [193], *Rice tungro spherical virus* in rice by editing the *eIF4G* gene [199], *Pepper mottle virus* (PepMoV) in tomato by editing the *eIF4E* gene [200]. In case of DNA viruses, resistance has been developed against *Tomato yellow leaf curl virus* in tomato plants by targeting the host susceptibility factor *Ty-5* (*SlPelo*) gene using a CRISPR/Cas9 system [201]. Thus, targeting host susceptibility factors through CRISPR tools is one of the best approaches for developing broad-spectrum and durable resistance against viruses. Further identification of novel host susceptibility factors will lead to widespread application of CRISPR for virus resistance.

## 9. Conclusions

Plant viruses continue to pose one of the most serious threats to global crop production. Their high mutation rates, broad host ranges, and dependence on insect vectors for transmission allow for rapid adaptation to new environments and hosts. In parallel, climate change is altering the ecology of host–virus–vector interactions, causing shifts in unpredictable patterns of disease outbreaks. Intensified global trade of planting materials and human interventions have further compounded the trans-boundary spread of plant viruses, frequently turning endemic outbreaks into epidemics or pandemics. Advances in genomics and molecular diagnostics, such as high-throughput sequencing technologies, have improved our understanding of virus diversity, evolution, and epidemiology and have enabled the simultaneous detection of novel viruses in mixed infections. Additionally, isothermal amplification assays and portable diagnostics are emerging as promising field-deployable tools for rapid detection of viruses. However, sample preparation, reagent stability, multiplexing capacity and field robustness remain obstacles to their widespread usage in high-throughput diagnostics. Rapid, sensitive, and specific diagnostic tests that differentiate viruses at the species level coupled with strict phytosanitary measures will be crucial to reducing the spread of plant viruses. Historical evidence shows that many viral epidemics were caused by the movement of infected planting materials through trade and germplasm exchanges, which underscores the importance of robust testing and certification systems. Therefore, early disease warning and timely intervention will depend on the development of reliable, multiplex, and field-deployable diagnostic platforms for real-time surveillance. In addition to detecting viruses before symptoms manifest, improved diagnostic technologies also aid in risk assessment and regulatory decisions (quarantine listings). Moreover, the identification of host susceptibility factors that are essential to the virus’s life cycle and its exploitation through genome editing tools are paving the way for durable resistance. Integrating these findings with modern breeding tools, like marker-assisted selection, has the potential to develop crop varieties with more stable and durable resistance to viruses. However, the success of such strategies depends on ongoing regular surveillance of virus populations to rapidly detect any resistance-breaking variants that can overcome resistance. Future research should focus on integrative approaches that combine multi-omics analyses and epidemiological modeling with weather forecasting data to better understand and predict virus emergence under changing conditions. Such integrated efforts will be critical to predicting emerging risks and developing climate-resilient and sustainable cropping systems in changing climate scenarios.

## 10. Future Prospects

The growing incidence of emerging viruses like CABYV, criniviruses, GPGV, BBWV-2, TSWV, and ToLCNDV reflects a fast-changing landscape of invisible invaders with expanding host ranges. Increasingly erratic disease patterns under changing climatic conditions add additional complexity to forecasting and the development of management strategies. Moreover, our knowledge of host cellular factors that affect viral replication and systemic movement is very limited. Though some host cellular factors have been identified, their precise function in viral pathogenesis remains elusive. Gaining insights into these aspects is essential to devising durable resistance strategies beyond R-gene-based approaches. Additionally, the way in which several viruses are transmitted remains largely unclear. For example, under controlled conditions, the eriophyid mite (*Colomerus vitis*) has been identified as a vector of GPGV. Nevertheless, the involvement of other vectors under field conditions and the mechanism governing virus acquisition, retention, and transmission have yet to be fully elucidated. The limited understanding of the mechanism by which environmental pressures drive host shifts, virus emergence, and vector dynamics highlight several key research priorities.

Going forward, integrated efforts are needed to (i) identify host factors that facilitate viral replication and pathogenicity; (ii) elucidate the molecular and ecological determinants of virus transmission, including seed-borne pathways; (iii) explore the diversity of vector species and the role of environmental factors on disease occurrence; and (iv) map the diversity and distribution pattern of emerging or novel viruses worldwide, accurately assess their impact on crops or economic yield and classify them as quarantine pests under EPPO’s A1 and A2 lists. Addressing these questions is essential to developing predictive models and sustainable management strategies under rapidly changing conditions. Given the rapid evolution of viruses, these efforts must be accompanied by continuous monitoring using modern diagnostic tools at plant quarantine stations to control the spread of viruses causing latent infections.

## Figures and Tables

**Figure 1:**
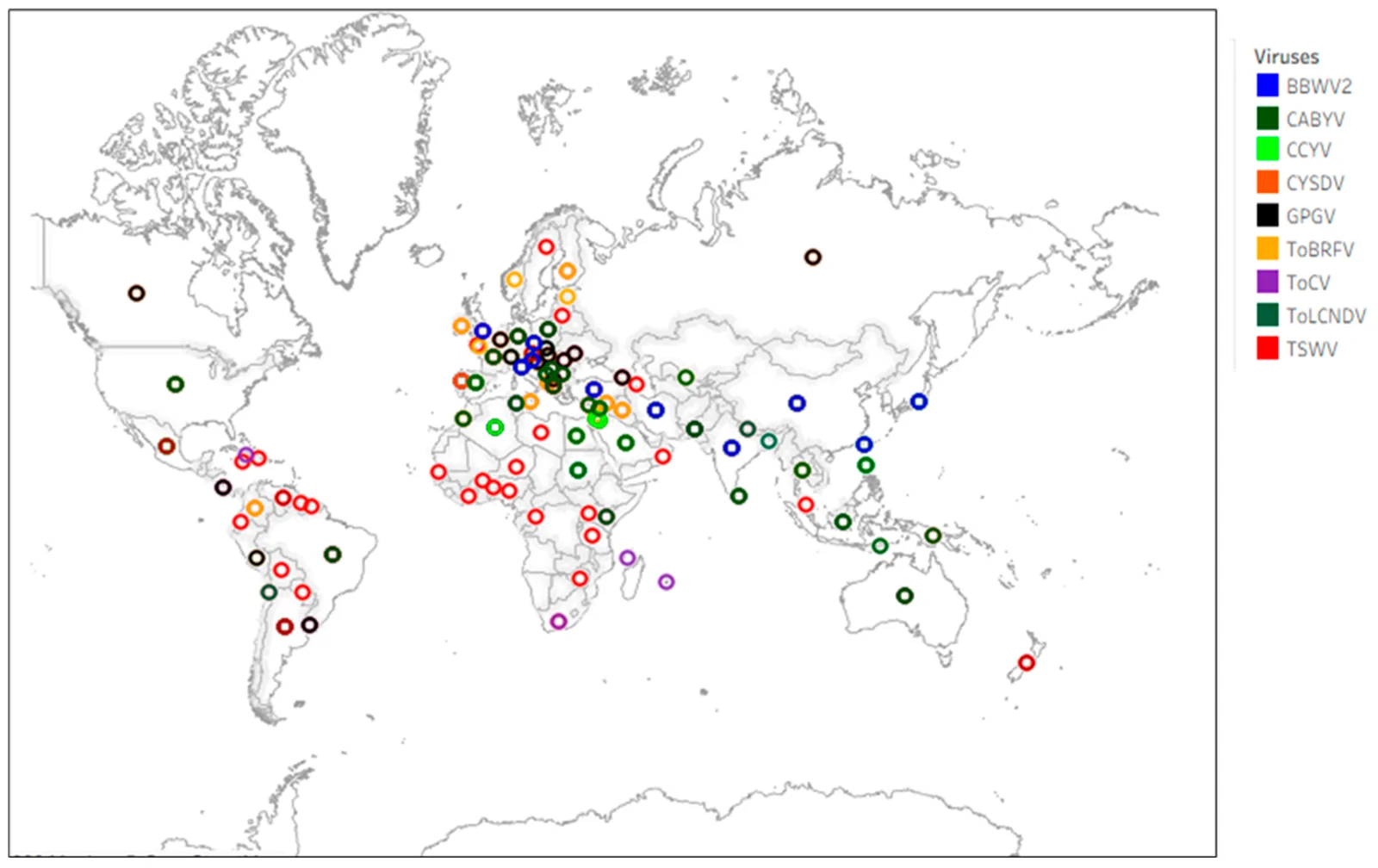
Global landscape of emerging and re-emerging plant viruses affecting diverse crops. (Map summarizing global distribution of major emerging viruses; see Figure 3 for details on virus distributions in each continent).

**Figure 2:**
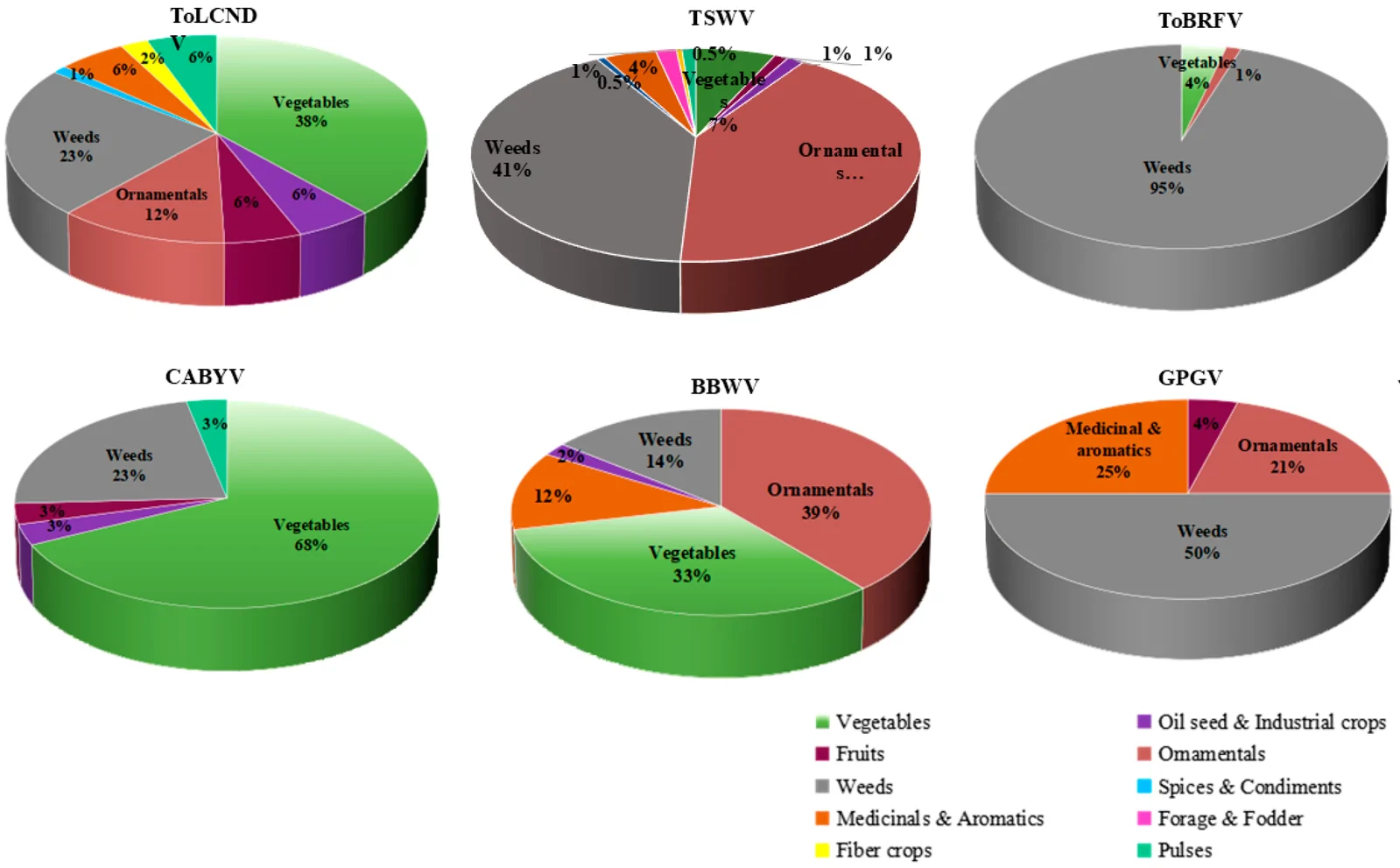
Host distribution of major plant viruses across crop categories. (Pie charts showing the proportional categories of hosts for key viruses: ToLCNDV, TSWV, ToBRFV, GPGV, BBWV-2, and CABYV).

**Figure 3:**
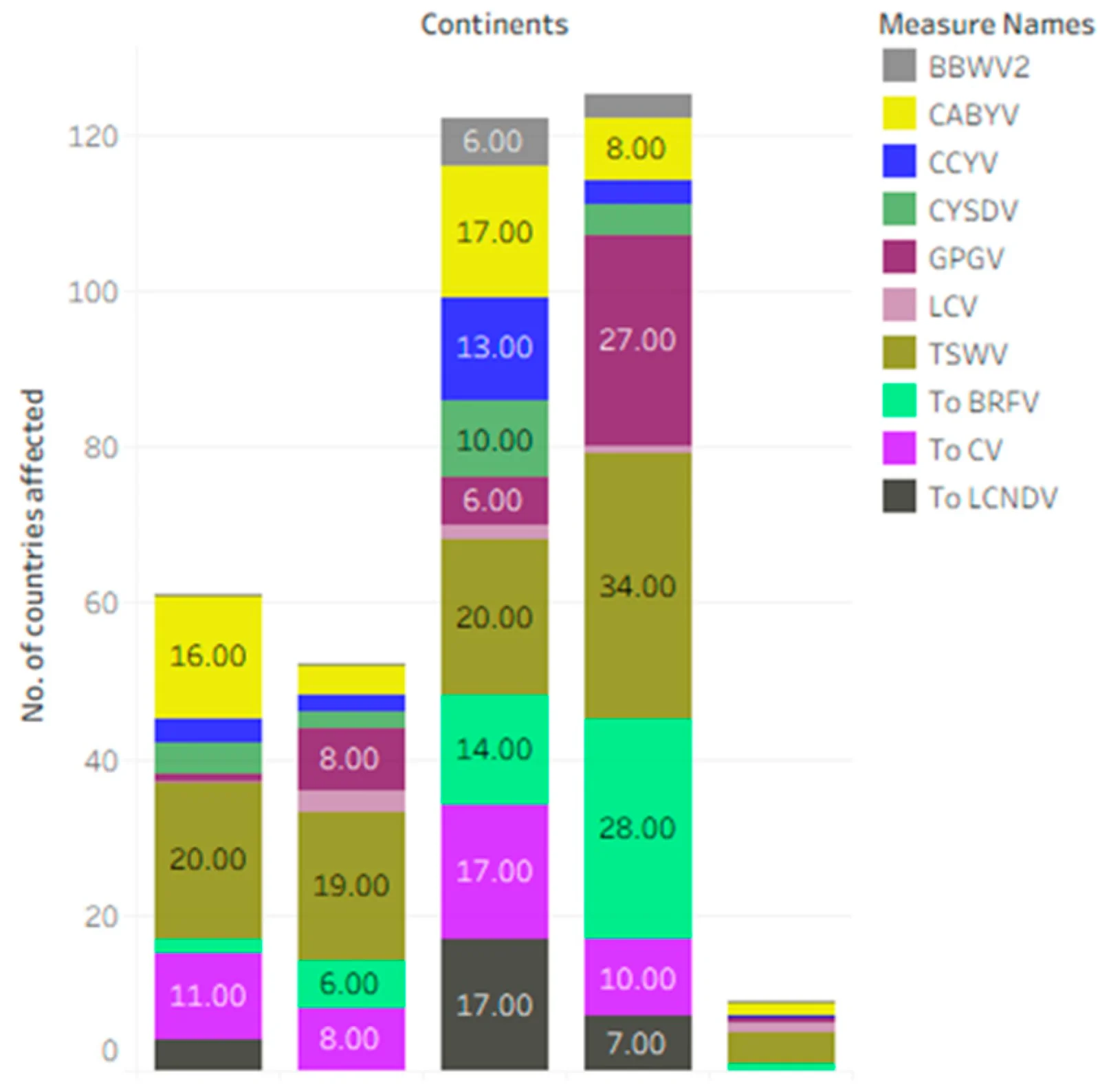
Distribution of plant viruses reported across countries in different continents. (Bar charts by continent; each color represents a different virus, and stacked values indicate the total number of countries reporting each virus in that continent).

**Figure 4:**
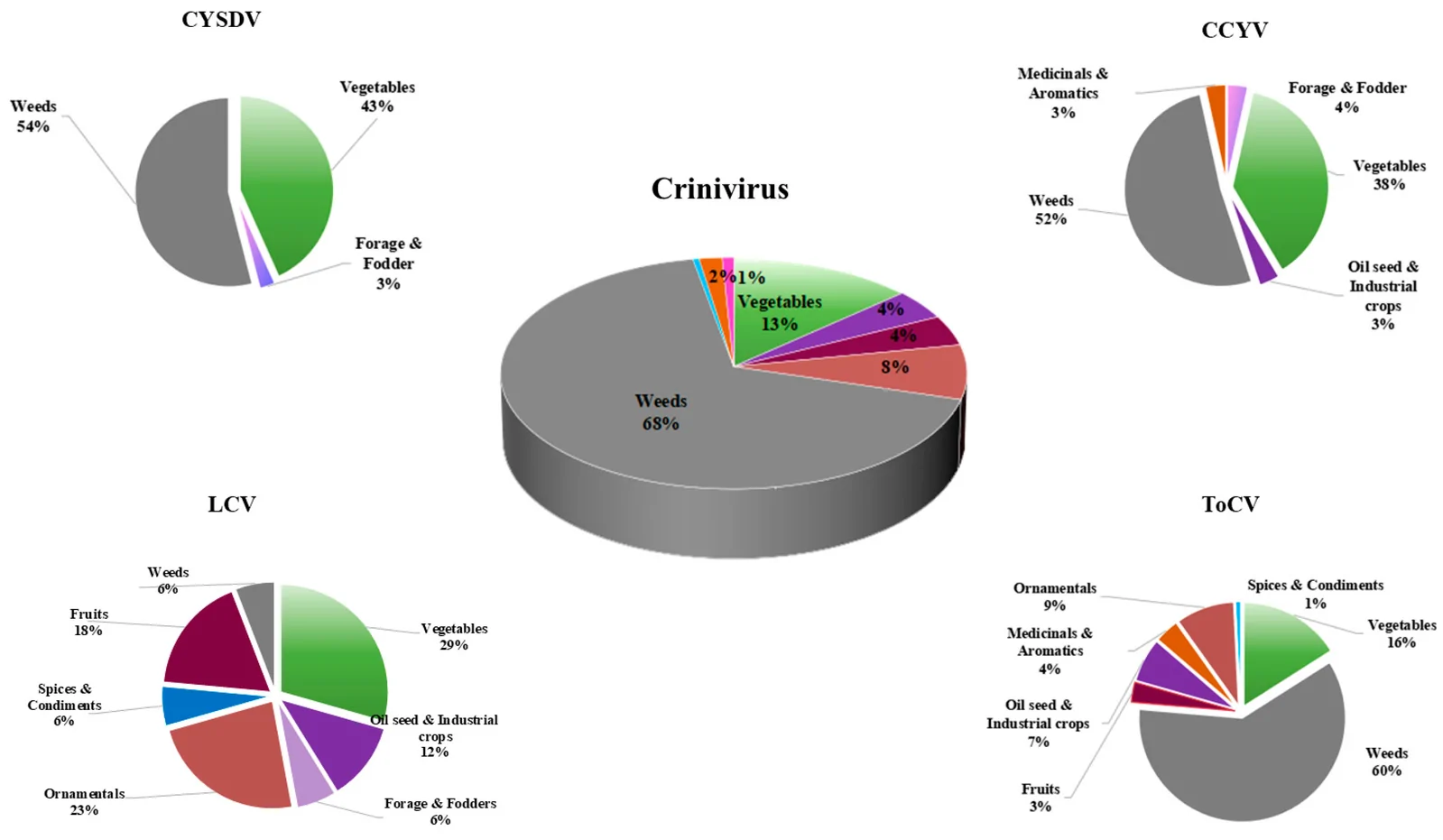
Total and species-wise contributions of four emerging Crinivirus species (CYSDV, CCYV, ToCV, and LCV) among reported host categories.

**Figure 5:**
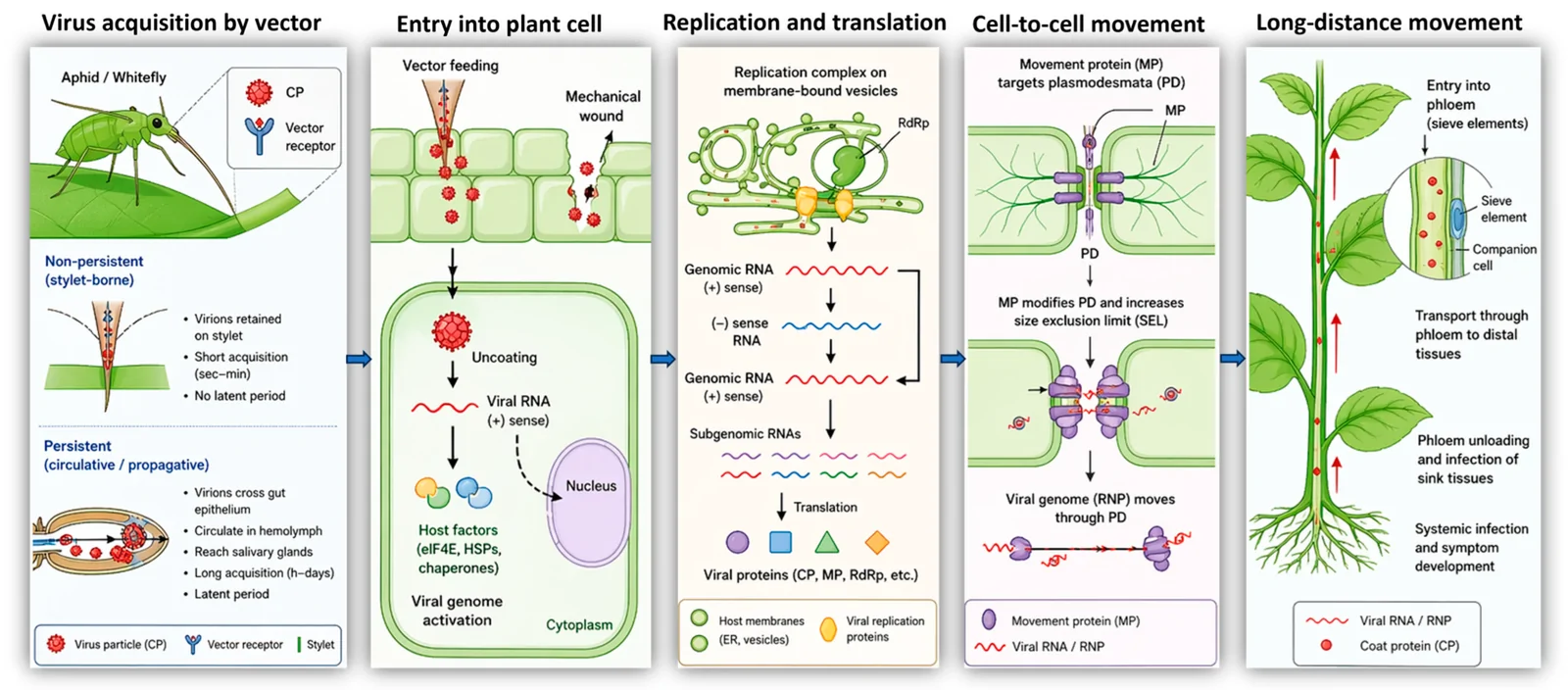
Molecular mechanisms of virus transmission and movement in plants. (This schematic illustrates key steps such as virus acquisition by a vector, entry into a plant cell, replication/translation in cells, cell-to-cell movement via plasmodesmata, and long-distance movement through the vascular system).

**Figure 6:**
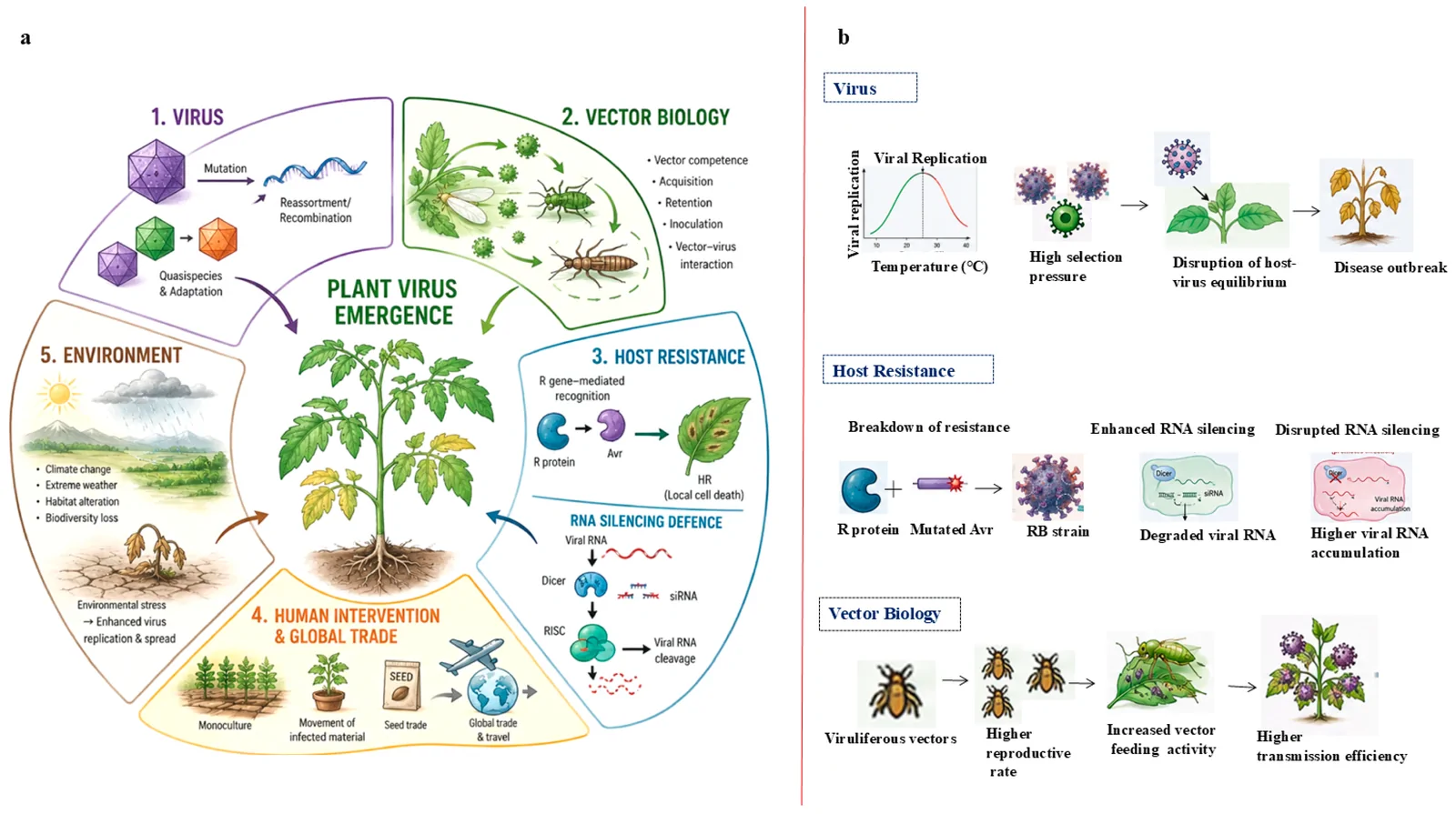
(**a**) Key determinants of plant virus emergence. (**b**) Climate change (temperature) drive virus emergence.

## Data Availability

The data supporting the findings of this study are available from the corresponding author upon reasonable request.

## Supplementary Materials

The following supporting information can be downloaded at: https://www.mdpi.com/article/10.3390/v18101144/s1, Table S1: Emerging and re-emerging viral diseases detected in economically important crops worldwide.
