# Supplementary material for: The Evolving Landscapes of Invisible Invaders: Global Insights on Plant Viruses and Vector Complexes
Source: Viruses. 2026 Oct 15;18(10):1144. doi: 10.3390/v18101144 (PMC13709711; doi:10.3390/v18101144)
Supplement: Supplementary file 1 [file viruses-18-01144-s001.zip › viruses-4266489-supplementary.pdf]

Table S1:Emerging and re-emerging viral diseases detected in economically important crops worldwide.

Crops	Symptoms	Country	Accession Number	Vector	References
**Tomato brown rugose fruit virus (ToBRFV), Genus Tobamovirus**					
Tomato	Chlorosis, brown necrosis	Spain	MT211630		Alfaro-Fernández et al. 2021, Plant Dis. 105, 515.
Tomato	Chlorosis	UK	MN182533		Skelton et al. 2019, New Dis. Rep. 40, 12.
Tomato	Mosaic	Palestine	MK165457		Alkowni et al. 2019, J. Plant Pathol. 101, 719–723.
Tomato	Mosaic, necrosis	Egypt	MT227801-02		Amer et al. 2020, New Dis. Rep. 41, 24.
Potato, tomato, Pepper	Mosaic or mottling,	Egypt	-		Khalifa et al. 2024, Fayoum J. Agric. Res. Dev. 38.
Tomato	Mosaic, deformation	Greece	MN815773		Beris et al. 2020, Plant Dis. 104, 2035.
Tomato	Yellowing, green grooves	Mexico	MK273183-MK273190		Cambrón-Crisantos et al. 2019, Rev. Mex. Fitopatol. 37, 185–192.
Chilli					
Tomato	Chlorotic, mosaic	Turkey	MK888980		Fidan et al. 2019, New Dis. Rep. 39, 18.
Tomato	Chlorotic, brown necrotic spots	Iran	OK075081		Ghorbani et al. 2021, New Dis. Rep. 44, e1204.
Tomato	Necrosis and ringspot	Norway	OK358628		Hamborg et al. 2022, Plant Dis. 106, 2004.
Tomato	Mosaic	Syria	MZ447794- 96		Hasan et al. 2022, Plant Dis. 106, 772.
Tomato	Light discoloured spots	Switzerland	OM305070		Mahillon et al. 2022, New Dis. Rep. 45, e12065.
Tomato	Chlorosis	Germany	MK133093-95		Menzel et al. 2019, New Dis. Rep. 39, 1.
Tomato<br>Sweet pepper	Mosaic, deformation	Italy	MK313803<br>MT188668		Panno et al. 2019, Plant Dis. 103, 1443.
Tomato	Brown spots, deformation	Saudi Arabia	MZ130501-03		Sabra et al. 2021, Plant Dis. 106, 1310.
Tomato	Mosaic, mottling	United States	MK109002		Ling et al. 2019, Plant Dis. 103, 1439.
Tomato	Brown rugose	China	MK905890		Yan et al. 2021, J. Integr. Agric. 20, 1871–1879.
Tomato	Brown rugose	Jordan	KT383474		Salem et al. 2016, Arch. Virol. 161, 503–506.
Solanum nigrum	Leaf narrowing, fruit discoloration	Jordan	OP009331		Salem et al. 2022, Plants 11, 2287.
Amaranthus retroflexus	Leaf narrowing	Jordan	OP009332		
Beta vulgaris	Leaf narrowing	Jordan	OP009333		
Chenopodiastrum murale	Leaf narrowing	Jordan	OP009334		
Corchorus olitorius	Leaf narrowing	Jordan	OP009335		
Erigeron canadensis	Leaf narrowing	Jordan	OP009336		
Malva parviflora	Leaf narrowing	Jordan	OP009337		
Oxalis corniculata	Leaf narrowing	Jordan	OP009338		
Portulaca oleracea	Leaf narrowing	Jordan	OP009339		
Taraxacum officinale	Leaf narrowing	Jordan	OP009340		

Veronica syriaca	Leaf narrowing	Jordan	OP009341		
Solanum elaeagnifolium	Leaf narrowing	Jordan	OP009342		
Tomato	Severe mosaic	Israel	KX619418		Luria et al. 2017, PLoS One 12, e0170429.
Tomato	Severe mosaic, blistering	Mexico	MK319944		Camacho-Beltran et al. 2019, Plant Dis. 103, 1440.
Pepper	Stunting, Puckering	Jordan	MK648157		Salem et al. 2020, Plant Dis. 104, 601.
Tomato	Yellow spots on the fruits	Palestine	MN013187-188		Jamous et al. 2022, Res. Plant Dis. 28, 98–107.
Tomato	leaf mosaic, leaf deformation	Iran	OM807073		Esmaeilzadeh et al. 2022, J Plant Pathol. 104, 893.
Sweet pepper	Mosaic	Syria Lebanon	OU600529-30		Abou Kubaa et al. 2022, J Plant Pathol. 104, 425.
Pepper	Browning and discoloration	Iran	ON528712		Bananej et al. 2024, J Plant Dis Prot. 131, 49.
Tomato	Mosaic and mottling	India	PP358541-49		Kavya et al. 2024, Australas. Plant Dis Notes. 19, 9.
Tomato	Chlorotic mosaic	Netherlands	-		Van De Vossenberg et al. 2020, PLoS One. 15, e0234671.
Tomato	Chlorotic, cupped leaves	Florida	MW322766		Dey et al. 2021, New Dis Rep. 44, e12028.
Tomato	Deformation and necrosis	Egypt	MT227801- 04		Amer et al. 2020, New Dis Rep. 41, 24.
Tomato	Mottlling, vein necrosis	China	OR437354		Guo et al. 2024, Plant Dis. 108, 2.
Tomato<br>Pepper	Leaf mottling, narrowing	Argentina	OR225613		Obregón et al. 2023, New Dis Rep. 48, e12203.
Tomato	Uneven ripening green blotchy	Argentina	PV289029-34		Ibañez et al. 2025, Viruses. 17, 533.
Tomato	Rough surfaces, blotchy pale brown spots	Colombia	PV350314-20		Sánchez et al. 2025, J Plant Pathol. 1.
Bell pepper	Mosaic, distortion	Mexico	PP477068-70		Mora-Ugalde et al. 2025, J Plant Pathol. 107, 711.
Convolvulus arvensis L.	No symptom	Sicily, Italy	OP150933		Cultrona et al. 2024, Plant Dis. 108, 827.
Polycarpon tetraphyllum L.	No symptom		OP194000		
Sweet pepper	Mosaic	Uzbekistan	OR543967		Akhmadaliev et al. 2025, Plant Dis. 109, 9.
Tomato	Mosaic and discoloration	Uzbekistan	OR501605		Bakhtiyorova et al. 2024, J Plant Pathol. 106, 779.
Persicaria perfoliata L.	Wrinkling, curling	China	CN0142961		Wang et al. 2025, Plant Dis. 109, 11.
Tomato	Malformation, shoestring	Lorestan	PQ760178-79		Baharvand et al. 2025, J Genet Resour. 11, 41.
Tomato	Uneven ripening and mottling	Jersey	PQ359482-85		Skelton et al. 2025, J Plant Pathol. 107, 1313.
Tomato	Mottling	France	MZ542763		Skelton et al. 2022, New Dis Rep. 45, 1.
Tomato	Mosaic, mottling	Peru	-		Rodriguez-Grados et al. 2024, MDPI Proc., 3rd Int Electron Conf Plant Sci.

Tomato	Mosaic, mottling	California	MT002973		Chanda et al. 2021, Virol. J. 18, 7.
Tomato	Severe mosaic	Albania	PQ643185		Magdalena et al. 2025, Phytopathol. Mediterr. 64, 77–86.
Tomato	Brown necrotic spots	Albania	OL763428-29		Orfanidou et al. 2022, J. Plant Pathol. 104, 855.
Tomato	Dwarf, deformation	Slovenia	ON045074-75		Vucurovic et al. 2022, New Dis. Rep. 45, e12079.
**Grapevine Pinot gris virus (GPGV), Genus Trichovirus**					
Grapevine	Stunting, deformation	Peru	PQ444178 PQ461689		Sánchez-Moncada etal. 2025, New Dis. Rep. 51, e70014.
Grapevine	Mottling, leaf deformation	Chile	MK029341-42		Zamorano et al. 2019, Plant Dis. 103, 1438.
Grapevine	Leaf mottling	Argentina	MT450471		Debat et al. 2020, J. Plant Pathol. 102, 1321.
Grapevine	Leaf mottling, deformation	Italy	LN606702-58		Saldarelli et al. 2015, Phytopathology 105, 555–563.
Grapevine	Asymptomatic	California	MW208104-09		Diaz-Lara et al. 2021, Plant Dis. 105, 2295–2298.
Grapevine	Asymptomatic	California	KT894101		Al Rwahnih et al. 2016, Plant Dis. 100, 1030.
Grapevine	Chlorotic mottling	United States	KT935465- 71		Angelini et al. 2016, Plant Dis. 100, 1254.
Grapevine	Chlorotic mottling,	Italy	KU312039		Gualandri et al. 2017, Eur. J. Plant Pathol. 147, 21–25.
Grapevine	Chlorotic mottling	Hungary	ON360686-99	-	Demian et al. 2022, Plants 11, 1830.
Chenopodium album	Chlorotic mottling	Hungary			
Asclepias syriaca	Chlorotic mottling	Hungary			
Ailanthus sp.	Chlorotic mottling	Hungary			
Rosa canina	Chlorotic mottling	Hungary			
Crataegus sp.	Chlorotic mottling	Hungary			
Sambucus sp.	Chlorotic mottling	Hungary			
Rubus sp.	Chlorotic mottling	Hungary			
Fraxinus sp.	Chlorotic mottling	Hungary			
Grapevine	Chlorotic mottling,	Hungary	GSE106240		Czotter et al. 2018, Front. Microbiol. 9, 122.
Grapevine	Mottling, leaf deformation	Australia	OQ198990-OQ199021		Kaur et al. 2023, Virol. J. 20, 211.
Grapevine	Chlorotic, mottling	Canada	PQ204588-PQ204610		Karki et al. 2025, Virology 603, 110360.
Grapevine	Mottling, deformations	Germany	PP348014-037		Messmer et al. 2024, Virol. J. 21, 178.
Grapevine	Zigzag growth of shoots,	Germany	KX522755		Reynard et al. 2016, Plant Dis. 100, 2545.
Grapevine	Mottling, deformations	Pakistan	KY747493-94		Rasool et al. 2017, Plant Dis. 101, 1958.
Grapevine	Shortened internodes, mosaic	Korea	AB731567		Cho et al. 2013, New Dis. Rep. 27, 2044–2058.
Grapevine	Mottling, deformations	Slovenia	HG738850 HG738852		Pleško et al. 2014, Plant Dis. 98, 1014.

Grapevine	Mottling, deformations	United Kingdom	MG983746		Silva et al. 2018, New Dis. Rep. 38, 2044–2058.
Grapevine	Leaf mottling	Czech Republic	KF686810		Glasa et al. 2014, Arch. Virol. 159, 2103–2107.
Grapevine	Mottling, deformations	Brazil	MF044018-19		Fajardo et al. 2017, Australas. Plant Dis. Notes 12, 45.
Grapevine	Mottling, deformations	Turkey	KR071599-KR071603		Gazel et al. 2016, Plant Dis. 100, 657.
Grapevine	Mottling, deformations	France	KM491305		Beuve et al. 2015, Plant Dis. 99, 293.
Grapevine	Mottling, deformations	Italy	KP204961-KP204981		Bertazzon et al. 2016, Arch. Virol. 161, 711–714.
Grapevine	Mottling, deformation	Canada	KU052861		Xiao et al. 2016, Plant Dis. 100, 1030.
Grapevine	Mottling, deformation	Italy	FR877530		Giampetruzzi et al. 2012, Virus Res. 163, 262–268.
Grapevine	Chlorotic mottling	Canada	KU194413		Poojari et al. 2016, Plant Dis. 100, 1513.
Grapevine	Chlorotic mottling	Japan	LC601601-LC601603		Abe & Nabeshima 2021, J. Plant Pathol. 103, 725.
Grapevine	Chlorotic mottling	Armenia	MN186552- 56		Eichmeier et al. 2020, Plant Dis. 104, 1000.
Grapevine	Mottling, deformations	Australia	KY243032-37		Wu & Habili 2017, Virus Genes 53, 935–938.
Grapevine	Mottling, deformations	Belgium	MN228488		Massart et al. 2020, Plant Dis. 104, 1879.
Grapevine	Mottling, deformations	Russia	KX522755		Navrotskaya et al. 2021, Viruses 13, 2432.
Grapevine	Mottling, deformations	Spain	KY404083		Ruiz-García and Olmos 2017, Plant Dis. 101, 1070.
**Cucurbit yellow stunting disorder virus (CYSDV), Genus Crinivirus**					
Satputia<br>Bitter gourd<br>Cucumber<br>Sponge gourd	Yellowing	India	OQ285912-15		Krishnan et al. 2022, Crop Prot. 158, 106013.
Ridge gourd<br>Snake gourd<br>Bottle gourd	Interveinal chlorosis	India	PP990497-500		Bharath et al. 2025, Crop Prot. 187, 106998.
Round melon<br>wild melon (Cucumis callosus)	Bleaching, blistering	India	PQ014400-02		Kumar et al. 2025, Virus Dis. 36, 93–96.
Melon<br>Cucumber	Yellowing	Spain	U67170		Celix et al. 1996, Phytopathology 86, 1370–1376.
Melon	Yellowing	Morocco	-	Bemisia tabaci	Desbiez et al. 2000, Plant Dis. 84, 596.
Cucumber	Yellowing	France	AY204220	Bemisia tabaci	Desbiez et al. 2003, Plant Dis. 87, 600.

Cucumber	Chlorotic mottling	Portugal	AF287474	Bemisia tabaci	Louro et al. 2000, Plant Dis. 84, 1156.
Cantaloupe (Cucumis melo)	Interveinal chlorosis	United states	KY404161-64	Bemisia tabaci	Gadhave et al. 2018, Plant Health Prog. 19, 9–10.
Watermelon	Stunting, deformation	Florida	EU596528	Bemisia tabaci biotype B	Polston et al. 2008, Plant Dis. 92, 1251.
Cucumber<br>Melon<br>watermelon	Yellowing	Spain	AY580884-985		Marco et al. 2005, J. Gen. Virol. 86, 815–822.
Melon	Severe stunting, yellowing	North America	AJ243000	Bemisia tabaci	Kao et al. 2000, Plant Dis. 84, 101.
Cucumber<br>Melon	Yellowing	Greece	-	Aphis gossypii<br>Myzus persicae	Boubourakas et al. 2006, Plant Pathol. 55, 276–283.
Pumpkin (C. maxima)<br>Cantaloupe (C. melo)<br>Cucumber<br>Winter squash (C. pepo)<br>Watermelon	Yellowing	Turkey	-		Yardimci et al. 2007, Australas. Plant Dis. Notes 2, 59.
Melon<br>Cucumber	Yellowing	Turkey	Y580985,<br>FJ492808	Trialeurodes vaporarium	Fidan et al. 2012, Proc. Xth EUCARPIA Meet. Genet. Breed. Cucurbitaceae, 755–762.
Melon	Interveinal chlorosis	California	LT992901	MEAM1 (biotype B)	Mondal et al. 2021, Plant Dis. 105, 3768.
Melon	Yellowing	Alabama	MZ073350	Bemisia tabaci	Mondal et al. 2022, Plant Dis. 106, 776.
Melon	Interveinal chlorosis	California	EF121768	B. tabaci biotype B	Kuo et al. 2007, Plant Dis. 91, 330.
Melon	Interveinal chlorosis	China	GU189240	B. tabaci biotype B	Liu et al. 2010, Plant Dis. 94, 485.
Zucchini<br>Squash	Leaf deformation	Spain	-	Bemisia tabaci	Gil-Salas et al. 2007, J. Virol. Methods 146, 45–51.
Alfalfa (Medicago sativa) lettuce (Lactuca sativa), snap bean (Phaseolus vulgaris)	Interveinal yellowing, leaf brittleness	California	NC004810	Bemisia tabaci	Wintermantel et al. 2009, Plant Dis. 93, 685–690.
**Cucurbit chlorotic yellows virus (CCYV), Genus Crinivirus**					
Cucumber<br>Melon<br>Watermelon	Yellowing	Japan	HG939521 -22	Bemisia tabaci	Orfanidou et al. 2014, Virus Res. 186, 120–129.
Cucumber	Yellowing	Pakistan	MN723535-36	-	Nouman et al. 2023, J. Plant Pathol. 105, 323–324.
Cucumber<br>Round melon<br>Muskmelon	Yellowing	India	OQ285908-10		Nagendran et al. 2023, Virology 587, 109876.
Melon,	Yellowing	Iran	KC559449-		Bananej et al. 2006, Plant Dis. 90, 526.

Cucumber<br>Watermelon<br>Squash			KC559451		
Melon	Yellowing, bleaching	Taiwan	KY618798-99	B. tabaci biotype B	Kang et al. 2017, J. Plant Med. 59, 54.
Watermelon	Yellow mottling, chlorosis	Florida	MZ325846-49		Hendricks et al. 2021, Plant Dis. 106, 339.
Cucumber	Interveinal chlorosis	Jordan	-		Salem et al. 2020, Plant Dis. 104, 3277.
Melon<br>Squash<br>Watermelon	Interveinal chlorosis	Mexico	EF21058 -59	Bemisia tabaci	Brown et al. 2007, Plant Dis. 91, 773.
Watermelon (Citrullus lanatus)<br>Zucchini (Cucurbita pepo)	Yellowing, vein clearing	Spain	OK562588-89	-	Alfaro-Fernández et al. 2022, Plant Dis. 106, 2006.
Cucumber	Interveinal chlorosis	Saudi Arabia	KT946810-14	*Trialeurodes vaporariorum*	Shakeel et al. 2018, Plant Dis. 102, 10–16.
Watermelon	Leaf crumpling, yellowing	United States	MW527460-62		Jailani, A.A.Ki et al., 2022
Squash (Cucurbita pepo)	Interveinal yellowing	United States	MW251341-42	Bemisia tabaci	Kavalappara et al. 2021a, Plant Dis. 105, 1862
Cantaloupe (Cucumis melo)	Interveinal chlorosis	Texas	MW584332-40	-	Hernandez et al. 2021, Plant Dis. 105, 3313.
Cucumber	Yellowing	South Korea	MW033300-01	Bemisia tabaci	Kwak et al. 2021, Plant Dis. 105, 6.
Lettuce	Blistering, chlorosis	India	OK018127	Bemisia tabaci	Kumar et al. 2023, Virus Dis. 34, 554–557.
Cucumber	Interveinal chlorosis	Spain	-		Chynoweth et al. 2021, Plant Dis. 105, 2258.
Cucumber	Yellowing	Egypt	KP204871-73		Amer 2015, Int. J. Virol. 11, 1–11.
Muskmelon (Cucumis melo)<br>Watermelon (Citrullus lanatus)	Yellowing	Philippines	MT859694-95	*Bemisia tabaci*	Chang et al. 2021, J. Plant Pathol. 103, 681–682.
Cucumber	Interveinal yellowing	Lebanon	JX014262-63	*Bemisia tabaci*	Abrahamian et al. 2012, Plant Dis. 96, 1704.
Cucumis melo	Chlorosis, brittleness	Taiwan	HM120250	*Bemisia argentifoli*i	Huang et al. 2010, Plant Dis. 94, 1168.
Cucumis melo	Mottling, vein chlorosis	Japan	AB523788-89	*Bemisia tabaci*	Okuda et al. 2010, Phytopathology 100, 560–566.
Melon,<br>Watermelon<br>Cucumber	Foliar chlorosis	China	GU721105<br>GU721118<br>GU721111	*Bemisia tabaci*	Gu et al. 2011, Plant Dis. 95, 73.
Muskmelon<br>Cucumber	Yellowing	Sudan	JF807053-55	*Bemisia tabaci*	Hamed et al. 2011, Plant Dis. 95, 1321.
Melon (*Cucumis melo*)	Interveinal yellowing	California	MH806867-68	*B. tabaci* MEAM1	Wintermantel et al. 2019, Plant Dis. 103, 778.
Melon	Interveinal chlorosis	California	MH477611	MEAM1 (biotype B)	Mondal et al. 2021, Plant Dis. 105, 3768.

Squash<br>Cucumber	Interveinal chlorosis	Alabama	MZ073347-49	*Bemisia tabaci*	Mondal et al. 2022, Plant Dis. 106, 776.
Hami melon (Cucumis melo cv. Xuelihong)	Yellowing	China	HQ148667	*Bemisia tabaci*	Zeng et al. 2011, Plant Dis. 95, 354.
Cantaloupe<br>Cucumber<br>Yellow squash	Interveinal chlorosis	United States	MW629379-81<br>MW685455-56<br>MW685461	*Bemisia tabaci*	Kavalappara et al. 2021b, Viruses 13, 988.
Cannabis sativa	Interveinal chlorosis	Israel	OR492252-53	*Bemisia tabaci* (MEAM1)	Gezovitch et al. 2024, Plant Pathol. 73, 47–56.
Zucchini	Yellowing	Algeria	MN529558-60	-	Kheireddine 2023, Doctoral Dissertation, Universitat Politècnica de València.
**Tomato chlorosis virus (ToCV), Genus Crinivirus Tomatichlorosis**					
Tomato	Yellowing, rolling	Argentina	PP066832	*Bemisia tabaci* complex, *Trialeurodes vaporariorum*<br>*T. abutiloneus*	Medina et al. 2025, J. Phytopathol. 173, e70064.
Sweet pepper (Capsicum annuum L.)	Interveinal chlorosis	Spain	-	*Bemisia tabaci*	Lozano et al. 2004, Plant Dis. 88, 224.
Cowpea (Vigna unguiculata)	Interveinal chlorosis	China	KX987242	*B. tabaci* (biotype Q)	Wang et al. 2018, Plant Dis. 102, 254.
Tomato	Chlorosis	China	KC887999	*B. tabaci*	Tang et al. 2017, Virol. J. 14, 169.
Potato	Mild chlorosis, purplish spots	Brazil	-	*B. tabaci* (MEAM1) and (MED)	Favara et al. 2025, Plant Pathol. 74, 1585–1592.
Amaranthus viridis<br>*Solanum americanum*	Interveinal chlorosis	Brazil	MN581161-62	*B. tabaci*	Souza et al. 2020, Trop. Plant Pathol. 45, 84–90.
Apple-of-Peru (AOP) (*Nicandra physaloides*)	Leaf rolling, interveinal chlorosis	Brazil	KY569401	*B. tabaci* (MEAM1)	Souza et al. 2019, Plant Dis. 103, 593.
Eggplant (*Solanum melongena*)	Yellowing, interveinal chlorosis	China	KR184676-77	*B. tabaci*	Zhou et al. 2015, Plant Dis. 99, 1657.
Tomato	Yellowing	Mayotte Island	AM748817-19	*B. tabaci*	Massé et al. 2008, Plant Pathol. 57, 388.
Tomato	Yellowing	Mauritius Island	AM884013-14	*B. tabaci*	Lett et al. 2009, Plant Dis. 93, 111.
Tomato	Yellowing	Japan	AB513442-44	*Trialeurodes vaporariorum*	Hirota et al. 2010, J. Gen. Plant Pathol. 76, 168–171.
Tomato	Interveinal yellowing	Sudan	JN411685	*B. tabaci*	Fiallo-Olivé et al. 2011, Plant Dis. 95, 1592.
Tobacco	Curling, interveinal chlorosis	Spain	KJ175084	*B. tabaci*	Fiallo-Olivé et al. 2014, Plant Dis. 98, 1162.

Tomato	Chlorosis, bunchy appearance	India	MN511714	-	Chaudhary et al. 2023, Virus Dis. 34, 56–75.
Tomato	Yellowing	Saudi Arabia	KJ433488-91	*B. tabaci*	Al-Saleh et al. 2014, Plant Dis. 98, 1590.
Tomato	Interveinal chlorosis	Korea	KP114532-33	*B. tabaci*	Lee et al. 2018, Plant Pathol. J. 34, 199.
Tomato	Interveinal chlorosis	Uruguay	KC626018	*B. tabaci* biotype Q T. vaporariorum	Arruabarrena et al. 2014, Plant Dis. 98, 1445.
Tomato	Yellowing	Tunisia	KJ739306-09	*B. tabaci*	Mnari-Hattab et al. 2014, New Dis. Rep. 30, 2044–0588.
Tomato	Interveinal yellowing	Jordan	KP713345-49	*B. tabaci*	Salem et al. 2015, Plant Dis. 99, 1286.
Tomato	Interveinal yellowing	Brazil	EU868927	*B. tabaci* biotype B	Barbosa et al. 2011, Trop. Plant Pathol. 36, 256.
Sweet pepper (Capsicum annuum	Interveinal yellowing	Brazil	-	*B. tabaci* biotype B	Barbosa et al. 2010, Plant Dis. 94, 374.
Sweet pepper (Capsicum annuum	Interveinal yellowing	Costa Rica	-	Trialeurodes vaporariorum	Vargas et al. 2011, Plant Dis. 95, 1482.
Ruta chalepensis	Interveinal yellowing	Costa Rica	-	*B. tabaci*	Solórzano-Morales et al. 2011, Plant Dis. 95, 497.
Phytolacca icosandra	Interveinal yellowing	Costa Rica	-	*B. tabaci*	
Plantago major	Interveinal yellowing	Costa Rica	-	*B. tabaci*	
Tomato	Leaf curl, yellowing	Lebanon	DQ234079	*B. tabaci*	Abou-Jawdah et al. 2006, Plant Dis. 90, 378.
Tomato	Interveinal chlorosis	Italy	AY048854	-	Accotto et al. 2001, Plant Dis. 85, 1208.
Tomato	Interveinal chlorosis	Mexico	DQ377368	*B. tabaci*	Alvarez-Ruiz et al. 2007, Plant Pathol. 56, 1043.
Solanum nigrescens	Interveinal chlorosis	Mexico			
Datura stramonium	Interveinal chlorosis	Mexico			
Tomato	Interveinal yellowing, necrotic flecks	Cyprus	AM158958	*B. tabaci* (biotypes A, B), *T. vaporariorum* *T. abutilonea*	Papayiannis et al. 2006, Plant Pathol. 55, 567.
Tomato	Interveinal chlorosis	Turkey	AY903448	*B. Tabaci* *T. vaporariorum*	Çevik & Erkış 2008, Plant Pathol. 57, 767.
Tomato	Interveinal chlorosis	Cuba	-	*B. tabaci* (biotypes B)	Martínez-Zubiaur et al. 2008, Plant Dis. 92, 836.
Tomato	Interveinal chlorosis	Israel	-	*B. tabaci*	Segev et al. 2004, Plant Dis. 88, 1160.
Potato	Mild chlorosis	Spain	-	*B. tabaci* (biotypes Q)	Fortes & Navas-Castillo 2012, Eur. J. Plant Pathol. 134, 81.
Tomato	Severe yellowing, chlorosis	Costa Rica	FJ809714	*T. vaporariorum*	Castro et al. 2009, Plant Dis. 93, 970.
Tomato	Interveinal yellowing, necrosis	Hungary	HQ444266	*B. tabaci* *T. vaporariorum* *T. abutilonea*	Bese et al. 2011, Plant Dis. 95, 363.

Tomato	Interveinal yellowing	Taiwan	-	*B. tabaci*	Tsai et al. 2004, Plant Dis. 88, 311.
Cucumber	Chlorosis, mottling	Brazil	MN172419-20	*B. tabaci* (biotypes Q)	Bello et al. 2020, Plant Dis. 104, 603.
Tomato	Interveinal yellowing	Brazil	OL449950	-	Keller et al. 2023, Ciência Rural 53, e20210883.
Teak (*Tectona grandis*)	Interveinal yellowing	Brazil	MH688047	-	Borges et al. 2019, Plant Dis. 103, 2704.
Tomato	Interveinal yellowing	Kenya	LC437668-71	*B. tabaci*	Kimathi et al. 2020, Sci. Afr. 7, e00286.
Potato	Leaf roll, interveinal chlorosis	Brazil	JQ288896-98	-	Freitas et al. 2012, Plant Dis. 96, 593.
Eggplant	Interveinal yellowing	Puerto Rico	KT727949-50	*B. tabaci* (MEAM1)	Fonseca-Boiteux et al. 2016, Plant Dis. 100, 867.
Scarlet eggplant	Interveinal yellowing	Puerto Rico	KT727954		
Tomato	Interveinal chlorosis	South Africa	KY471129	*B. tabaci* (MED)	Moodley et al. 2019, PLoS One 14, e0220298.
Tomato	Leaf brittleness	Florida	DQ234671-75	*B. tabaci* (biotype B)<br>*T. vaporariorum*	Wintermantel et al. 2006, Plant Dis. 90, 814–819.
Tomato	Interveinal yellowing	Portugal	AF234029	*B. tabaci*	Louro et al. 2000, Eur. J. Plant Pathol. 106, 589–592.
Tomato	Leaf bronzing, interveinal chlorosis	Korea	-	*B. tabaci*	Kil et al. 2015, Eur. J. Plant Pathol. 142, 419–426.
Conyza canadensis	Interveinal chlorosis	Korea	-	*B. tabaci*	
Erigeron annuus	Interveinal chlorosis	Korea	-	*B. tabaci*	
Sonchus asper	Interveinal chlorosis	Korea	-	*B. tabaci*	
Youngia japonica	Interveinal chlorosis	Korea	-	*B. tabaci*	
Trigonotis peduncularis	Interveinal chlorosis	Korea	-	*B. tabaci*	
Cardamine flexuosa	Interveinal chlorosis	Korea	-	*B. tabaci*	
Cerastium glomeratum	Interveinal chlorosis	Korea	-	*B. tabaci*	
Stellaria media	Interveinal chlorosis	Korea	-	*B. tabaci*	
Chenopodium album	Interveinal chlorosis	Korea	-	*B. tabaci*	
Ipomoea hederacea	Interveinal chlorosis	Korea	-	*B. tabaci*	
Quamoclit coccinea	Interveinal chlorosis	Korea	-	*B. tabaci*	
Vicia angustifolia var. Segetilis	Interveinal chlorosis	Korea	-	*B. tabaci*	
V. tetrasperma	Interveinal chlorosis	Korea	-	*B. tabaci*	
Phytolacca americana	Interveinal chlorosis	Korea	-	*B. tabaci*	
Mazus pumilus	Interveinal chlorosis	Korea	-	*B. tabaci*	
Solanum americanum	Interveinal chlorosis	Korea	-	*B. tabaci*	
S. nigrum	Interveinal chlorosis	Korea	-	*B. tabaci*	
Tomato	Mottling, yellowing	China	KY967219	-	Lyu et al. 2023, Viruses 15, 1617.
Potato	Mottling, yellowing	China	KY967227	-	

Tomato	Chlorosis	China	KY471138-39	-	Yang et al. 2025, Virology 604, 110447.
Tomato	Chlorosis	Nigeria	MF459657 -58	*B. tabaci*	Mohammed et al. 2018, Plant Dis. 102, 257.
Tomato	Interveinal chlorosis	Tunisia	KC156613-33	-	Gharsallah et al. 2015, Phytoparasitica 43, 87–96.
Physalis ixocarpa	Interveinal yellowing	Portugal	EF187609	*B. tabaci* (biotypes Q)	Trenado et al. 2007, Eur. J. Plant Pathol. 118, 193–196.
P. peruviana	Interveinal yellowing	Portugal	EF187610	*B. tabaci* (biotypes Q)	
Tomato	Interveinal yellowing	Spain	AF215817-18	*B. tabaci* (biotypes Q)	Navas-Castillo et al. 2000, Plant Dis. 84, 835–837.
Tomato	Interveinal yellowing	France	-	*T. vaporariorum*	Dalmon et al. 2005, Plant Dis. 89, 1243.
Tomato	Interveinal yellowing	Brazil	EU868927	MEAM1, MED, NW1, NW2, *T. abutiloneus* *T. vaporariorum*	Bampi et al. 2019, Plant Pathol. 68, 1019–1024.
Tomato	Interveinal yellowing	China	KC709509-10	-	Zhao et al. 2014, J. Phytopathol. 162, 627–634.
Tomato	Yellowing	France	EU625350	-	Jacquemond et al. 2009, Plant Pathol. 58, 210–220.
Tomato	Interveinal chlorosis	Georgia	HQ879840-47	*B. tabaci* (biotype B)	Sundaraj et al. 2011, Plant Dis. 95, 881.
**Lettuce chlorosis virus (LCV), Genus Crinivirus L*actuca chlorosis***					
Cannabis sativa	Interveinal chlorosis	Israel	MK747245-46	*B. tabaci*	Hadad et al. 2019, Viruses 11, 802.
Calopogonium mucunoides	Wrinkled leaves, mild mosaic	Australia	OQ377539-40	*B. tabaci*	Filardo et al. 2023, Australas. Plant Dis. Notes 18, 9.
Tobacco	Vein chlorosis, leaf distortion	China	KX685958-59	*B. tabaci*	Zhao et al. 2018, Arch. Virol. 163, 751–754.
Tomato	Chlorosis	China	KX158863	*B. tabaci*	Zhang et al. 2017, Plant Dis. 101, 846.
Chinese cabbage (*B. rapa* subsp. pekinensis)	Chlorosis	China	NC_012909		Li et al. 2025, Plant Dis. 109, 2233.
Bean	Intervenal mottling	Spain	KC602375-76	*B. tabaci*	Ruiz et al. 2014, Plant Dis. 98, 857.
Sugarbeet	Yellowing	California	-	*B. tabaci* (biotype B)	Wisler et al. 1997, Plant Dis. 81, 550.
Papaya	Mosaic, distortion	United States	KY271955-56	-	Alabi et al. 2017, Plant Dis. 101, 1094–1102.
Periwinkle (*Catharanthus roseus)*	Interveinal yellowing	China	KY430285-86	-	Tian et al. 2018, Plant Dis. 102, 462.
Cilantro (Coriandrum sativum)	Yellow blotch	California	MN395647-55	-	Alabi et al. 2020, Plant Dis. 104, 630–633.
Periwinkle	Mosaic , leaf malformation	Brazil	MN216391-92	-	Favara et al. 2020, Plant Dis. 104, 1263.
Passiflora auriculata *P. alata*	Yellowing, mosaic	Brazil	MN564795-98	-	Vidal et al. 2021, J. Plant Pathol. 103, 443–447.

Lettuce (*Lactuca sativa*)		California	FJ380118-19	*B. tabaci* (biotype A, B)	Nida'M et al. 2009, Virology 390, 45–55.
**Broad Bean Wilt Virus 2, genus Fabavirus**					
Marigold (Tagetes erecta)	mosaic	China	MW322809		Dong et al. 2021, Plant Dis. 105, 3767
Sesame (Sesamum indicum L.)	mosaic, chlorotic mottle	China	KY446012		Wang et al. 2017, Plant Dis. 101, 1556
ribwort plantain plants (Plantago lanceolata)	mosaic and mottling	Iran	PP845352		Mehrvar et al. 2024, J. Plant Pathol. 106, 1863
pepper plants (Capsicum annuum)	concentric rings, dark bumps on fruits	Slovenia	EF405826		Mehle et al. 2008, Plant Pathol. 57
Echinacea purpurea	necrosis, leaf roll	China	JX070674		Li et al. 2012, Plant Dis. 96, 1232
Gynura procumbens (longevity spinach)	chlorotic local lesions	Korea	KX686589<br>KX686590		Kwak et al. 2017, Plant Dis. 101, 514
Chinese yam (Dioscorea opposita Thunb.)	mosaic, stunting, mottle	Korea	KT246495<br>KT246496		Kwon et al. 2016, Plant Dis. 100, 538
Eggplant (Solanum melongen)	stunting, necrosis	China	-		Rui et al. 2020, J. Plant Pathol. 102, 543
Balsam (Impatiens balsamina)	blotches, mosaic	China	OQ857921<br>OQ857922		Qiu et al. 2024, Plant Dis. 108, 232
Achyranthes bidentata	mosaic and yellowing	Korea	MH447988<br>MH447989		Kwon and Seo 2019, Plant Dis. 103, 165
Chinese cabbage (Brassica rapa L. var. chinensis	mosaic leaf	China	KY886225		Cai et al. 2017, Plant Dis. 101, 1559
Tropaeolum majus	chlorotic spots and mosaic	China	KX698095		Wei and Li 2017, Plant Dis. 101, 1332
Bupleurum chinense	mosaic, distortion	China	FJ485684<br>FJ485685		Sui et al. 2009, Plant Dis. 93, 844
Mirabilis jalapa	shrinking, yellowing	China	OM416039<br>ON677747<br>ON677748		Chen et al. 2023, Plant Dis. 107, 1957
Ranunculus asiaticus cv. Elegance plants	leaf deformation and mosaic	Italy	KJ715961		Minutolo et al. 2016, Plant Dis. 100, 235
Perilla frutescens	mosaic, vein clearing	China	MN380789		Xia et al. 2020, Plant Dis. 104, 3085
Leonurus sibiricus	mosaic, yellowing	Korea	KM076648<br>KM076649		Seo et al. 2014, Plant Dis. 98, 1748
Commelina communis		China	MZ571836<br>MZ571837		Gang et al. 2023, Bangladesh J. Bot. 569–574
Catharanthus roseus	Mosaic, vein yellowing	India	OR826802<br>OR827717		Abbas et al. 2025, ACS Omega 10, 32433–32443

Veronica gentianoides	severe distortion and mottling	UK	MF429951		Fowkes et al. 2017, New Dis. Rep. 36
Foxglove (Digitalis spp. cv. Saltwood Summer)	Mottle, necrosis	UK	-		Mumford et al. 2006
Vicia faba	Severe mosaic, vein clearing	Korea	KC625492<br>KC625506		Kwak et al. 2013, Plant Pathol. J. 29, 397
Pisum sativum	Severe mosaic, vein clearing	Korea	KC625498<br>KC625512		Kwak et al. 2013, Plant Pathol. J. 29, 397
Spinacia oleracea	Severe mosaic, vein clearing	Korea	KC625505<br>KC625518		Kwak et al. 2013, Plant Pathol. J. 29, 397
Capsicum annuum (Red Pepper)	Severe mosaic, vein clearing	Korea	JX183221<br>JX183222		Kwak et al. 2013, Plant Pathol. J. 29, 397
sweet pepper (Capsicum annuum)	mosaic	Japan	AB023484		Kobayashi et al. 2003, J. Gen. Plant Pathol. 69, 320–326
Pepper (Capsicum annuum)	systemic mottling	Czech Republic			Svoboda and Leisova-Svobodova 2013, Plant Dis. 97, 1261
Spinacia oleracea	yellowing, wrinkling mottling	Korea	PV102464<br>PV102465		Zhao et al. 2025, Int. J. Mol. Sci. 26, 5946
Pepper	wilting, vein clearing	Turkey	-		Balsak et al. 2023, Physiol. Mol. Plant Pathol. 126, 102037
Chenopodium album	mottling	China	MN786954<br>MN786955		Li et al. 2020, CABI Databases 51, 446–453
Chinese yam (Dioscorea opposita Thunb.)	vein-clearing, rugosity	Japan	AB207244		Kondo et al. 2005, J. Gen. Plant Pathol. 71, 441–443
Gynura procumbens (longevity spinach)	mosaic and yellowing	Korea	KX686589-90		Kwak et al. 2017, Plant Dis. 101, 514
**Cucurbit aphid-borne yellows virus (CABYV)**					
Bitter gourd	Yellowing	India	MN843966		Kumar et al. 2021, VirusDisease 32, 183–185.
Cucumber	Yellowing	India	MN843966		Kumar et al. 2021, VirusDisease 32, 183–185.
Melon, zucchini	Yellowing	France<br>Greece<br>Turkey	-	*M. persicae*<br>*Aphis gossypii*	Lecoq et al. 1992, Plant Pathol. 41, 749–761.
Cucumber,	Yellowing				
Zucchini	Yellowing				
Squash	Yellowing				
Bitter gourd	Dark green mottling	India	MK182528-29	*Aphis gossypii*	Sangeetha et al. 2019, Plant Dis. 103, 1441.
Teasel gourd	Green vein banding	India	KY711340	-	Suveditha et al. 2017, New Dis Rep. 36, 7.
Bitter gourd	Green vein banding	India	MF281974	-	
Pumpkin	Yellowing	Europe	KX398663	*Aphis gossypii*	Knierim et al. 2010, Plant Pathol. 59, 991–1002.
Cucumber	Yellowing	Thailand	KF815679	*Aphis gossypii*	

Capsella bursa-pastroris	Yellowing, thickening	Taiwan	GU324097-104	*Aphis gossypii*	
Squash	Yellowing	Spain	JF939812-14	*A. gossypii*	Kassem et al. 2007, Plant Dis. 91, 232–238. Kassem et al. 2013, Phytopathology 103, 1188–1197.
Melon (*Cucumis melo*)	Severe yellowing	Tunisia	EF187338-46	*A. gossypii* *M. persicae*	Mnari-Hattab et al. 2009, Plant Dis. 93, 1065–1072.
Cucumber (*C. sativus*)	Severe yellowing				
Zucchini (*Cucurbita pepo*)	Severe yellowing				
Squash (*C. maxima*),	Severe yellowing				
Watermelon (*Citrullus lanatus*),	Severe yellowing				
Ware cucurbit (*Ecballium elaterium*)	Severe yellowing				
Watermelon	Yellowing	Spain	MW051362-63	*A. gossypii* *M. persicae*	Rabadan et al. 2021, Plant Pathol. 70, 1476–1485.
Squash	Yellowing				
Cucumber	Yellowing	Germany	JX262071	-	Menzel et al. 2020, New Dis Rep. 41, 2044–0588.
Musk mallow (*Abelmoschus moschatu)*	Yellowing, midvein clearing	India	OP455156	-	Verma et al. 2024, Plant Dis. 108, 540.
Pumpkins (*Cucurbita pepo*)	Yellowing	Serbia	HQ202745	*A. gossypii*	Vucurovic et al. 2011, Plant Dis. 95, 1035.
Pumpkins (*Cucurbita pepo*)	Chlorotic spots	Poland	MT384365-66	-	Minicka et al. 2020, Plants 9, 820.
Melon	Yellowing, mosaic	Korea	KR231959-63	-	Kwak et al. 2018, Plant Pathol. J. 34, 532.
Pumpkins (*Cucurbita pepo*)	Yellowing	Turkey	MK129536	*A. gossypii*	Arslan et al. 2020, Fresen. Environ. Bull. 29, 7239–7245.
Cucumber	Yellowing with green-vein banding	Indonesia		*A. gossypii* *M. persicae*	Listihani et al. 2020, J. Gen. Plant Pathol. 86, 219–223.
Cucumis melo	Yellowing	Indonesia	LC384060-61	*A. gossypii* *M. persicae*	Damayanti et al. 2021, IOP Conf. Ser.: Earth Environ. Sci. 948, 012023.
Momordica charantia					
Melon	Yellowing	United States	-	*A. gossypii* *M. persicae*	Lemaire et al. 1993, Plant Dis. 77, 1169.
Marrows					
Melon (Cucumis melo var. Cantalauop)	Interveinal mottling	Italy	EF029114-15	-	Tomassoli et al. 2007, Plant Pathol. 56, 720.
Melon	Yellowing	Korea	LC060147	*A. gossypii*	Choi et al. 2015, Plant Pathol. J. 31, 371.
Melon	Yellowing	Pakistan	MH141556	-	Ahsan et al. 2020, J. Plant Pathol. 102, 563–564.
Squash	Yellowing, mosaic	Turkey	MT219979	-	Topkaya et al. 2020, Zemdirbyste-Agriculture 107, 353–358.
Melon	Yellowing, mosaic	Turkey	MT219983		
Pumpkin	Yellowing, mosaic	Turkey	MT219984		
Cucumber	Yellowing, mosaic	Turkey	MT219988		
Bottle gourd	Yellowing, mosaic	Turkey	MT219992		

Ridge gourd (*Luffa acutangula*)	Yellowing	Pakistan	PP112273	-	Asad et al. 2022, Pak. J. Bot. 54, 467–474.
Round melon *(Praecitrullus fistulosus*)	Yellowing	India	OP455157		Tripathi et al. 2024, J. Plant Pathol. 106, 279.
Squash	Yellowing	China	EU244321	-	Shang et al. 2009, Virus Res. 145, 341–346.
Cucumber	Yellowing	China	EU262624		
Muskmelon	Yellowing	China	EU244315		
Wax gourd	Yellowing	China	EU000534		
Watermelon	Yellowing	China	EU091150		
Bitter gourd	Yellowing	China	EU091148		
Spong gourd	Yellowing	China	JQ700306		
bitter melon	Yellowing	China	JQ700305		
Cucumber	Yellowing	Japan	GQ221224	-	Momma et al., 2025
Cucumber	Interveinal chlorosis	Saudi Arabia	LM653105-06	-	Al Saleh et al. 2015, Plant Dis. 99, 894.
Squash	Interveinal chlorosis	Saudi Arabia			
Cucumber	Yellowing	Lebanon	-	*A. gossypii*	Abou-Jawdah et al. 1997, Plant Dis. 81, 1331.
Muskmelon	Yellowing	Lebanon			
Squash (*Cucurbita pepo*)	Yellowing	Egypt	FR717145-51		Omar etal. 2012, Phytoparasitica 40, 177–184.
Cucumber	Thickening of older leaves	Turkey		*A. gossypii* *M. persicae*	Yardımcı et al. 2007, Aust. Plant Dis. 2, 59.
Squash	Thickening of older leaves	Turkey			
Watermelon	Thickening of older leaves	Turkey			
Muskmelons	Thickening of older leaves	Turkey			
Zucchini (*Cucurbita pepo*)	Severe mosaic, Chlorosis	Poland	MK059479	*A. gossypii* *M. persicae*	Zarzyńska-Nowak et al. 2019, Plant Dis. 103, 1047.
Squash (Cucurbita pepo)	Yellowing	Czech Republic	HM771269-73		Svoboda et al. 2011, Plant Dis. 95, 220.
Pumpkin (*Cucurbita pepo*)	Yellowing, vein thickening	Oklahoma	MG590258-62		Khanal et al. 2018, Plant Dis. 102, 1046.
Melon	Yellowing	Brazil	-	*B. tabaci* MEAM1	Costa et al. 2020, Plant Pathol. 69, 1042–1050.
Bitter gourd (*Momordica charanti*a)	Yellowing, thickening of basal leaves	Srilanka	MH802612	-	Abeykoon et al. 2018, New Dis Rep. 38, 22.
Spiny gourd (*Momordica dioica*)	Yellowing, thickening of basal leaves				
Cucumber	Yellowing	Philippines	HQ700823-24	-	Knierim et al. 2014, Arch. Virol. 159, 1459–1465.
Angled luffa (*Luffa acutangula*)	Yellowing	Philippines			
Ivy gourd (*Coccinia*	Yellowing	Thailand	KF427707		

grandis)			KF427704		
Cucumber	Yellowing	Thailand			
Muskmelon (*Cucumis melo*)	Yellowing	Uzbekistan	KF427706		
Watermelon	Yellowing	India	MN688219		Krishnan et al. 2022, Physiol. Mol. Plant Pathol. 120, 101840; Krishnan et al. 2022, Crop Prot. 158, 106013.
Ivy gourd	Yellowing	India	MN688220		
Squash	Yellowing	India	OQ285916		
Ridge gourd (*Luffa acutangula*)	Yellowing, green vein-banding	Iran	OQ351953-59		Pouraziz et al. 2023, Viruses 15, 1714.
Bitter gourd (*Momordica charantia*)	Yellowing, green vein-banding	Iran	OQ703990-OQ704000		
Lettuce	Yellowing	Greece	LN865080		Lotos et al. 2016, Arch. Virol. 161, 431–436.
Spinach	Yellowing	Greece	LN865083		
Cucumis melo	Yellowing	Greece	FN600343		
Vicia faba	Chlorosis	Turkey	KY112798		Buzkan et al. 2013, Arch. Virol. 158, 881–885.
Chickpea	Yellowing	Sudan	MG933685-86	*A. gossypii*	Kumari et al. 2018, Plant Dis. 102, 2048.
Groundnut	Interveinal chlorosis	Kenya	-	*A. gossypii*<br>*M. persicae*	Mabele et al. 2023, Plant Health Prog. 24, 79–82.
Passion Fruit	Blisters, mosaic	Brazil	OP909804-12	*Bemisia tabaci*	Vidal et al. 2023, Viruses 15, 410.
Bitter gourd	Yellowing	India	-	*A. gossypii*	Bose et al. 2025, Arch. Phytopathol. Plant Prot., 1–16.
Passion Fruit	Mosaic, mottling	China	MN119288	-	Zhang et al. 2020, Plant Dis. 104, 601.
Melon	Yellowing, mosaic	Spain	PP869188	-	de Moya-Ruiz et al. 2025, Plant Pathol. 74, 270–282.
Watermelon	Yellowing, mosaic	Spain	PP869202		
Cucumber	Yellowing	Papua New Guinea	MG780352		Maina et al. 2018, Genome Announc. 6, e00162-18.
Cucumber	Yellowing	East Timor	KY617826	*A. gossypii*<br>*M. persicae*	Maina et al. 2017, Genome Announc. 5, 10-1128.
Pumpkin	Yellowing	Slovenia	MN145451-57		Mehle et al. 2020, Plant Dis. 104, 599.
Cucumber	Yellowing	Slovenia			
Musk melon	Yellowing	Slovenia			
Water melon	Yellowing	Slovenia			
Cucumber,	Yellowing	Cyprus		*Bemisia tabaci*	Papayiannis et al. 2005, J. Phytopathol. 153, 530–535.
Zucchini,	Yellowing	Cyprus			
Melon	Yellowing	Cyprus			
Watermelon	Yellowing	Cyprus			

Pumpkin	Interveinal yellowing	Bulgaria	MK671010		Radeva-Ivanova et al. 2022, Plant Dis. 106, 2538.
Cucumber	Interveinal yellowing	Bulgaria	MK671014		
Melon	Leaf curling, yellowing	Mediterranean Region		Aphis craccivora *M. Persicae* *Bemisia tabaci*	Radouane et al. 2021, Phytopathol. Mediterr. 60, 493–520.
Cucumber	Leaf curling, yellowing				
Watermelon	Leaf curling, yellowing				
Squash	Leaf curling, yellowing				
Cucumis sativus	Yellowing, thickening	Iran	KF425566	*A. gossypii* *M. persicae*	Vafaei et al. 2017, Iran J. Biotechnol. 15, 289–295.
Bryonia dioca	Yellowing, thickening	Iran	KX432189		
Sysimbrium irio	Yellowing, thickening	Iran	KX432190		
Citrullus colocynthis	Yellowing, thickening	Iran	KX432191		
Papaver rhoeas	Yellowing, thickening	Iran	KX432192		
Capsella bursa-pastoris	Yellowing, thickening	Iran	KX432193		
Eruca sativa	Yellowing, thickening	Iran	KX432199		
Snake gourd,	Chlorotic spots	India	OQ921128	-	Verma et al. 2024, Plant Dis. 108, 540.
Ridge gourd	Chlorotic spots	India	OQ921127		
Bittergourd,	Chlorotic spots	India	OQ921126		
Ash gourd	Chlorotic spots	India	OQ921125		
Sponge gourd	Chlorotic spots	India	OQ921129		
Summer squash	Interveinal mottling	Montenegro	-	-	Zindovic et al. 2017, J. Plant Pathol. 99, 299.
Melon	Interveinal mottling	Montenegro			
Cushaw (Cucurbita moschata)	Yellowing with severe mosaic	China	EF063705	-	Xiang et al. 2008, Plant Pathol. 57, 390.
Squash (*Cucurbita pepo*)	Yellowing with severe mosaic	China	DQ973124		
Cucumber (*Cucumis sativus*)	Yellowing with severe mosaic	China	EF063706		
Suakwa vegetable sponge (*Luffa cylindrical*)	Yellowing with severe mosaic	China	EF063707		
Melon	Yellowing	Mediterranean	OM948834	*A. gossypii* *M. persicae*	Rabadán et al. 2023, Phytopathology 113, 1761–1772.
Zucchini	Yellowing	Mediterranean	OM948857		
Zucchini (*Cucurbita pepo*)	Yellowing	Morocco	MH414922		Aarabe et al. 2018, Australas. Plant Dis. Notes 13, 29.
Melon	Yellowing, necrosis of leaf	Spain	EU660581-86	*Bemisia tabaci*	Juarez et al. 2013, Ann. Appl. Biol. 162, 362–370.
Squash	Yellowing, necrosis of leaf	Spain			
Pumpkin	Yellowing, necrosis of leaf	Spain			
Watermelon	Yellowing, necrosis of leaf	Spain			
Cucumber	Yellowing, necrosis of leaf	Spain			

Ecballium elaterium	Yellowing	Spain	NC_003688	*A. gossypii*<br>*M. persicae*	Maachi et al. 2022, J. Virol. 96, e00421-22.
